# Current Research in Polypharmacology for Cancer Treatment Using Dual-Target Histone Deacetylase Inhibitors

**DOI:** 10.3390/ijms27156604

**Published:** 2026-07-24

**Authors:** Pavel Yudaev, Yulia Aleksandrova, Margarita Neganova

**Affiliations:** Nesmeyanov Institute of Organoelement Compounds, Russian Academy of Sciences, Vavilova St., 28, Bld. 1, Moscow 119991, Russia; yudaevpavel5@gmail.com (P.Y.); yulia.aleks.97@mail.ru (Y.A.)

**Keywords:** cancer, inhibitor, dual-target agent, histone deacetylase, anaplastic lymphoma kinase, DNA methyltransferase, hydroxamic acid

## Abstract

The review covers research on dual-target antitumor agents over the past five years. One of the targets is histone deacetylases (HDACs), while the second potential target is a protein group located both on the membrane surface (phosphatidylinositol 3-kinase (PI3K), anaplastic lymphoma kinase (ALK), receptor tyrosine kinase (AXL), tyrosine protein kinase (HER2), FMS-like tyrosine kinase (FLT3), and vascular endothelial growth factor receptor (VEGFR2)) and in the nucleus (serine/threonine protein kinase Wee1, DNA methyltransferase (DNMT), dual-specificity phosphatase (CDC25A), an enzyme from the cyclin-dependent kinase family (CDK9), dual-specificity tyrosine-serine/threonine kinase (DYRK2), and BET family proteins (BRD4, BD1, and BD2)). This review presents the results of studies on the inhibitory activity of various HDAC isoforms and other enzymes, as well as in vitro cytotoxicity studies on both neoplastic and healthy cells. It also includes selectivity studies, in vivo experiments (changes in tumor volume in mice) and oral bioavailability assessments. The review also describes the chemical structures of several dual-target agents and identifies the molecular fragments responsible for inhibiting different targets. Based on the studies reviewed in this paper, it can be concluded that some dual inhibitors have superior in vitro cytotoxicity and exhibit selectivity towards some tumor cells compared to monofunctional reference compounds. These findings may be useful for molecular design in the field of polypharmacology, with the aim of developing new dual-target molecules that exhibit improved antitumor activity and selectivity towards neoplastic cells.

## 1. Introduction

Cancer is a complex disease that is the leading cause of death worldwide and a major challenge for healthcare systems [1,2]. Treating malignant tumors is difficult due to the complex nature of their development. Key factors involved include genetic and epigenetic alterations [3] as well as changes in signaling pathways and the immune system [4].

Currently, the main treatment methods for oncological diseases include chemotherapy, radiation therapy, surgery, and immunotherapy. These methods are often used in combination [5]. However, they have significant limitations due to their high toxicity to healthy tissues and the development of drug resistance. Conventional first-line chemotherapy drugs, such as cisplatin and paclitaxel, have nonspecific systemic effects that can cause multiple side effects and limit the acceptable doses [6]. Neoplastic cell resistance to cisplatin is due to effective repair of DNA damage through nucleotide excision, mismatch repair and homologous recombination [7]. The role of epigenetic modifications in cisplatin resistance has also been confirmed [7]. Therefore, the development of new drugs that are more effective and less toxic at specific times of administration is a crucial area of modern pharmacology.

**Figure 1:**
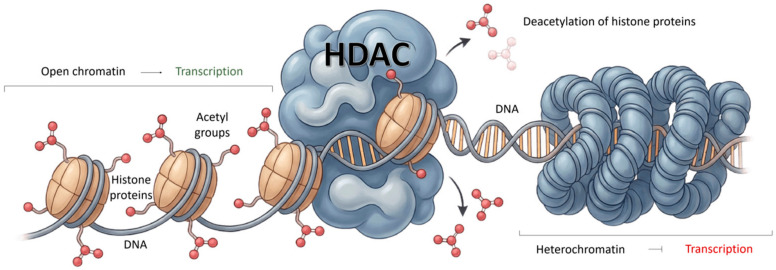
Epigenetic regulation of the transmission of genetic information with the participation of the enzyme histone deacetylase (HDAC).

Epigenetic modulation, especially involving histone deacetylases (HDACs) and histone acetylases, is implicated in the progression of tumors of various localizations [8], in particular glioblastoma [9], melanoma [10], and lymphoma [11]. HDACs play a key role in chromatin condensation and transcriptional silencing, altering gene expression by eliminating acetyl groups from histones and non-histone proteins (Figure 1) [12].

Given the significant role of epigenetic regulation in cancer development, epigenetically targeted therapies represent a promising approach to the treatment of malignant tumors. These therapies are effective both as stand-alone treatments and in combination with other modern treatments [13].

HDAC inhibitors (HDACis) have demonstrated promising antitumor activity [14]. Several HDACis, including vorinostat, panobinostat and belinostat, have been approved for clinical use in the treatment of cutaneous T-cell lymphoma, multiple myeloma and relapsed or refractory peripheral T-cell lymphomas [15].

However, FDA-approved HDACis lack demonstrable selectivity for a class (three classes of zinc-dependent HDACs) or an isoform (11 isoforms), which may lead to off-target effects in patients. Currently, there are two popular approaches to developing HDACis for cancer therapy: the isoform-selective inhibitor strategy and the dual-inhibitor strategy. Many HDACis with outstanding performance in preclinical or clinical studies have been developed using these two distinct approaches [16].

Since we discussed the first strategy in a previous review published in 2025 [17] and in other researchers’ reviews [18,19,20,21], this paper will focus on publications over the past five years in the field of dual-target drug design. As shown in earlier reviews [22,23], combined inhibition of multiple oncogenic signaling pathways involved in cancer progression using multiple chemotherapeutic agents often produces synergistic or additive effects and can reduce the likelihood of developing drug resistance. However, compared to combination strategies (two or more chemotherapy drugs), a single agent that simultaneously modulates two drug targets typically causes fewer adverse reactions, is less toxic [24], and exhibits a predictable pharmacokinetic profile [25]. In addition, the use of a single dual-target drug avoids the risk of drug interactions [26]. In this regard, the study of dual-target drugs that affect both epigenetic and signaling mechanisms in cancer has become a promising new approach to overcoming drug resistance in cancer treatment.

According to the Scopus database, between 2020 and 2025, there were 294 articles published on dual-targeting inhibitors, including 34 reviews and 255 research papers. The remaining articles were book chapters and abstracts. The reviews examined the biological activity of various compounds, such as HDAC inhibitors, tubulin [27], HDAC/PI3K, HDAC/CDK [27], and LSD1/HDAC [28]. In this paper, we will focus on the cytotoxicity of these dual-acting compounds, which reduce HDAC overexpression and target both the tumor cell membrane and nucleus.

## 2. Cell Membrane Receptors

### 2.1. Dual PI3K/HDAC Inhibitors

Phosphatidylinositol 3-kinases (PI3Ks) are a family of enzymes that play a crucial role in intracellular signaling pathways in response to external stimuli [29]. At the beginning of the 21st century, researchers discovered that components of the PI3K/AKT signaling pathway are often dysregulated in various types of cancer [30], including hepatocellular carcinoma and lymphocytic leukemia [31]. PI3Ks phosphorylate phosphatidylinositol 4,5-bisphosphate (PIP2) to generate phosphatidylinositol 3,4,5-triphosphate (PIP3), activating protein kinase B (AKT). This activation leads to a series of events that regulate cellular functions [32]. Aberrant activation of the PI3K pathway due to genetic mutations can contribute to cell transformation, tumor growth, and drug resistance [33].

**Figure 2:**
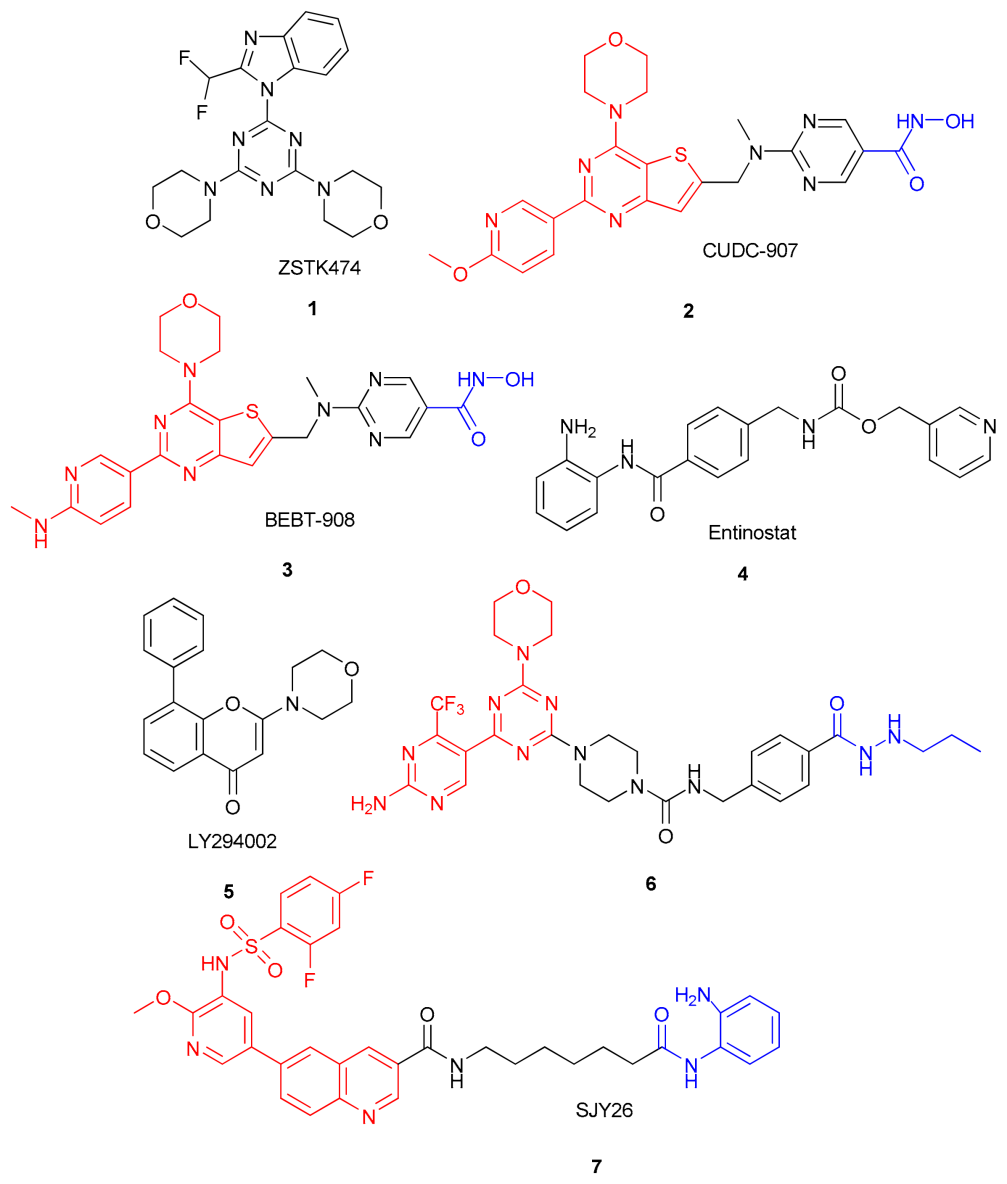
Structures of PI3K inhibitors and dual PI3K/HDAC inhibitors. The ALK-inhibiting pharmacophore is shown in red, and the zinc-binding group of the HDAC inhibitor is shown in blue. The fragment of the molecule responsible for PI3K inhibition is marked in red, and HDAC inhibition is marked in blue.

The class of PI3K inhibitors, including ZSTK474 (compound **1**, Figure 2), has demonstrated nanomolar IC_50_ values for α, β, γ, and δ isoforms in preclinical studies. This compound is currently undergoing clinical trials in patients with metastatic solid tumors [34]. However, the efficacy of monotherapy with these inhibitors has been limited due to the lack of selectivity [35]. Resistance to these inhibitors is mediated by the activation of alternative targets, such as AKT and CDK [36,37]. Compensatory signaling pathways, which can be blocked by HDAC inhibitors, may also contribute to resistance [38,39].

Dual PI3K/HDAC inhibitors have shown great potential as novel anticancer agents. For instance, fimepinostat (CUDC-907, **2**) and ifupinostat (BEBT-908, **3**) have completed clinical trials (Figure 2), demonstrating manageable side effect profiles and long-lasting efficacy in patients with relapsed/refractory diffuse large B-cell lymphoma [40].

In 2025, the antitumor activity of CUDC-907 (**2**), a compound with the chemical structure shown in Figure 2, was studied in relation to other tumor types, particularly metastatic medulloblastoma (MB) of group 3 (G3) [41]. CUDC-907 inhibits the overexpression of the MYC oncogene by blocking the HDAC and PI3K signaling pathways, leading to G0/G1 cell cycle arrest through the MYC-P21/P27-CDK-cyclin axis. The researchers found that CUDC-907 is a more potent inhibitor of G3 MB growth than the available HDAC inhibitor entinostat (**4**, Figure 2) and PI3K inhibitor LY294002 (**5**, Figure 2). This makes CUDC-907 a promising candidate for further development as a therapeutic agent for G3 MB.

The combination of CUDC-907 and cisplatin resulted in an enhanced G0/G1 arrest. Additionally, the combination with radiation therapy inhibited DNA repair and increased DNA damage.

The authors of [42] synthesized a new PI3K/HDAC inhibitor (compound **6**, Figure 2) by combining the morpholino-triazine pharmacophore from the structure of the PI3K inhibitor ZSTK474 (**1**) and the hydrazide moiety from a previously synthesized HDAC inhibitor responsible for the inhibition of HDAC1–3. The introduction of the propylhydrazide moiety into the molecule was due to its selectivity for HDAC1–3 isoforms [43]. Based on molecular docking results, the carbonyl group of the hydrazide moiety formed a chelate complex with the zinc ion Zn^2+^, and the hydrazide group formed hydrogen bonds with Phe144 and Tyr298 of HDAC3. Meanwhile, the substituted aminopyrimidine moiety formed salt bridges with Gly826 and Asp911 in PI3Kα.

The synthesized compound demonstrated simultaneous nanomolar inhibitory activity against HDAC and PI3Kα/β/δ/γ, showing selectivity for the HDAC1–3 isoforms (Table 1).

A study on the anti-tumor activity of a compound revealed its high cytotoxicity against various types of cancer cells, including biphenotypic B-myelomonocytic leukemia MV4-11, lymphoma Jeko-1, human leukemia HL60, and breast cancer MCF-7. The compound also displayed selectivity for cancer cells compared to normal human hepatocytes HL7702.

Western blot analysis showed a significant inhibition of AKT phosphorylation, an increase in the levels of acetylated histones H3 and H4, a decrease in the levels of Bcl-xL and procaspase 3, and an increase in the levels of γH2AX and cleaved caspase 3. These findings support the conclusion that the PI3K/AKT/mTOR signaling pathway has been blocked.

Another research group investigated compound SJY26 (compound **7**, Figure 2) as a dual-target PI3K/HDAC inhibitor. It contains a quinoline-linked sulfonamide–pyridinium moiety as the PI3K-inhibition pharmacophore and an *o*-aminobenzamide moiety as the zinc-binding moiety for the HDAC inhibitor. The structure of SJY26 was generated by combining the pharmacophore groups of vorinostat (or SAHA) and omipalisib. The choice of the *o*-aminobenzamide fragment as the zinc-binding group was based on its better pharmacokinetic properties and lack of genotoxicity compared to hydroxamic acid fragments [45]. Molecular docking established the formation of hydrogen bonds between the quinoline ring nitrogen and HDAC1 residues Tyr17 and Phe141 and between the sulfanilamide oxygen and Ser275 of PI3Kα.

Compound SJY26 showed inhibitory activity against PI3K in the nanomolar range and against HDAC1 at the micromolar level (Table 1). The cytotoxic effect of SJY26 was excellent against Jurkat T-cell leukemia, K562 human myeloid leukemia, MCF-7 breast adenocarcinoma, and PC9R human lung adenocarcinoma tumor cells, as shown by flow cytometry. This study revealed the induction of cell cycle disruption in the G2/M phase, with an increase in the G2/M population from 12.8% to 34.2%. Apoptosis analysis showed an increase in apoptotic cells from 7.41% to 11.1% after treatment with 2.5 μM SJY26 and up to 15.9% after treatment with 5 μM SJY26. Western blotting showed a decrease in AKT (Thr308) phosphorylation and an increase in histone H3 acetylation, but the study had limitations, including a lack of data on HDAC1–3 concentrations, isoform selectivity of HDAC1–3, and tumor cell specificity compared to healthy cells.

In addition, before proceeding with clinical trials, it would be beneficial to conduct additional pharmacokinetic studies, in vivo efficacy assessments, and modeling of lung and liver cancer metastases in mice for the dual-target PI3K/HDAC inhibitors being evaluated.

### 2.2. Dual ALK/HDAC Inhibitors

Anaplastic lymphoma kinase (ALK) is a protein kinase that belongs to the tyrosine kinase family and is a membrane protein. It is abnormally activated in various types of cancer, including non-small cell lung cancer [46], neuroblastoma [47], breast cancer [48] and thyroid cancer [49].

Currently, clinically approved ALK tyrosine kinase inhibitors include crizotinib (first-generation), ceritinib, alectinib, brigatinib (second-generation), and, finally, lorlatinib (third-generation). Unfortunately, patients treated with these ALK inhibitors for 1–2 years almost inevitably experience drug resistance due to ALK gene amplification or L1196M, G1269A, and G1202R mutations in the ALK domain [50]. These mutations alter the structure of the domain, making it difficult for the ALK inhibitor to bind.

A 2020 study found a synergistic effect of ALK and HDAC inhibitors, specifically crizotinib or alectinib and SAHA against PF240-PE lung adenocarcinoma cells [51]. Because HDACs are involved in regulating various pathways, such as TGF-β signaling and microRNA activity, this synergistic effect may be explained by the resensitization of ALK inhibitors through the downregulation of epithelial–mesenchymal transition [52].

**Figure 3:**
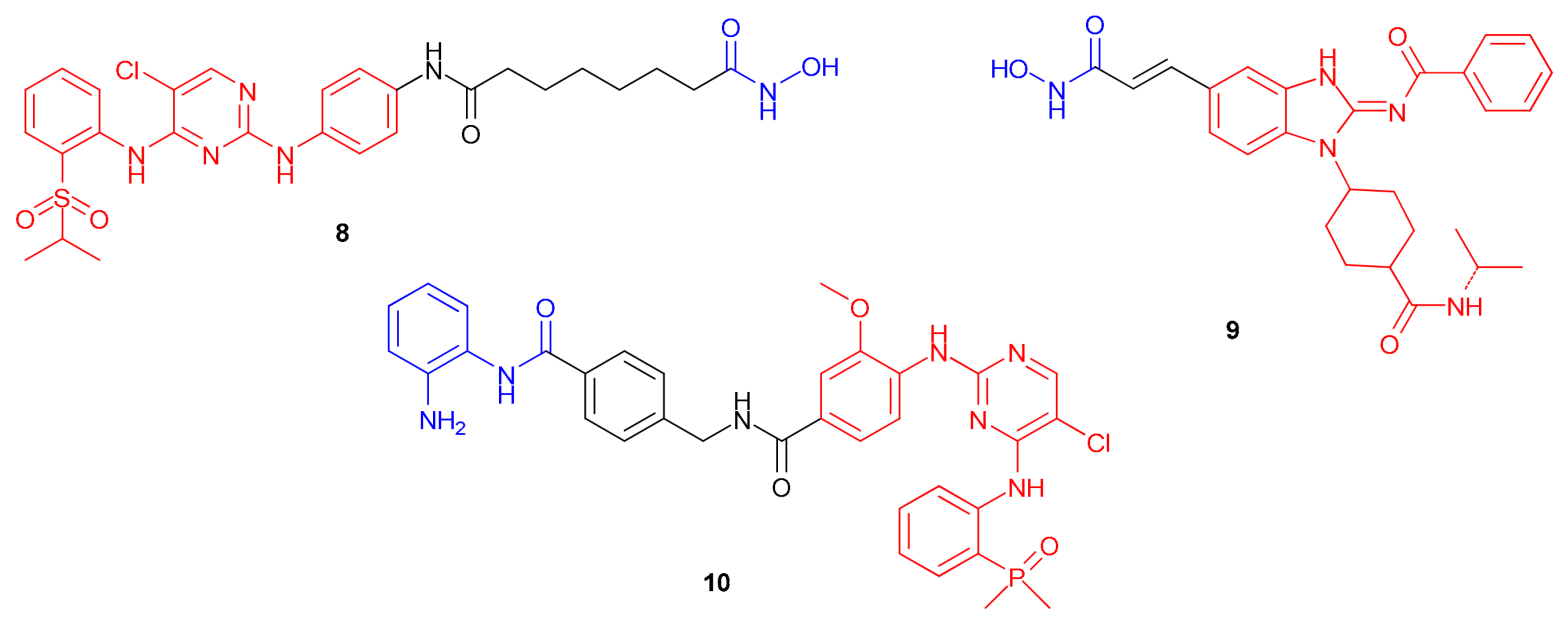
Structures of dual ALK/HDAC inhibitors. The ALK-inhibiting pharmacophore is shown in red, and the zinc-binding group of the HDAC inhibitor is shown in blue.

In a recent study, Pan et al. synthesized a dual ALK/HDAC inhibitor (compound **8**, Figure 3) based on a 2,4-pyrimidinediamine derivative by combining the structures of ceritinib and SAHA [53].

N4-(2-(isopropylsulfonyl)phenyl)-N2-phenylpyrimidine was chosen as the ALK inhibitor, and hydroxamic acid was selected as the zinc-binding group for the HDAC inhibitor. Molecular docking showed the interaction of the 2-aminopyrimidine with M1199 through a hydrogen bond, which is crucial for binding to ALK. The sulfonyl oxygen formed an H-bond with K1150’s amino group, and the hydroxamic acid created two coordinate bonds with Zn^2+^ at the HDAC2 active site. The resulting compound demonstrated high inhibitory activity against ALK, ALK^L1196M^, HDAC1, HDAC2, and HDAC6 at nanomolar concentrations (Table 2). Compared to ceritinib, it showed ~10-fold greater activity against the ALK^G1202R^ mutation associated with ceritinib resistance. Molecular docking identified three additional hydrogen bonds between the hydroxamate and R1202 and G1121.

The antiproliferative activity of compound **8** on HepG2, MDA-MB-231, A549, H2228, SK-N-BE(2), and SH-SY5Y cell lines was comparable to that of SAHA and ceritinib (IC_50_ ~1 μM; Table 2). In ALK-dependent SK-N-BE(2) and SH-SY5Y cells (F1174L) and H2228 cells (EML4-ALK), compound **8** showed superior activity compared to ceritinib and crizotinib. At a concentration of 0.4 μM, it inhibited the migration of MDA-MB-231 and SH-SY5Y cells by 3.7% and 2.8%, respectively. These cell lines exhibited a dose-dependent increase in apoptosis and acetylated H3 levels (a marker for HDAC1/2/3 inhibition), and a decrease in ALK, p-AKT, and p-ERK phosphorylation.

However, a potential drawback of the dual ALK/HDAC inhibitor mentioned above is its low water solubility and poor oral bioavailability. This was confirmed after oral administration of a 10 mg/kg dose in Sprague–Dawley rats. This is likely due to the presence of a hydrophobic linker, the SAHA fragment. To improve the bioavailability of this ALK/HDAC inhibitor, researchers [54] proposed a conjugate between an orally available ALK inhibitor and a benzimidazole core developed by Amgen Inc. in 2012 [55]. Additionally, they suggested combining this ALK inhibitor with the HDAC inhibitor pracinostat (compound **9**), as shown in Figure 3.

The resulting compound demonstrated the ability to inhibit both ALK^wt^ and the ALKL1196M mutant and HDAC6 in an enzymatic assay. Selective inhibition of the HDAC6 and HDAC8 isoforms was also observed compared to HDAC1 and HDAC11 (Table 2). Furthermore, the compound inhibited the proliferation of several tumor cell lines, including ALK-dependent human non-small cell lung cancer H2228 cells.

**Table 2:** In vitro data for ALK/HDAC dual-target inhibitors.

Compound	IC_50_ Against ALK/HDAC, nM	IC_50_, μM Against Cells	References
**8**	ALKwt 2.1 ALKL1196M 1.7 ALKG1202R 0.4 HDAC1 7.9 HDAC2 9.3 HDAC6 4.4	HepG2 0.64 ± 0.07 MDA-MB-231 0.46 ± 0.06 A549 0.46 ± 0.08 SK-N-BE2 0.05 ± 0.01 SH-SY5Y 0.11 ± 0.02 H2228 0.13 ± 0.05	[53]
SAHA	HDAC1 12 HDAC2 12 HDAC6 10	HepG2 1.43 ± 0.17 MDA-MB-231 0.61 ± 0.09 A549 0.59 ± 0.02 SK-N-BE2 0.77 ± 0.11 SH-SY5Y 0.73 ± 0.04	
Ceritinib	ALKwt < 1.0 ALKL1196M 0.25 ALKG1202R 3.3	HepG2 4.08 ± 0.27 MDA-MB-231 1.82 ± 0.63 A549 2.16 ± 0.10 SK-N-BE2 2.66 ± 0.20 SH-SY5Y 0.37 ± 0.10 H2228 1.98 ± 0.13	
**9**	ALKwt 16 ALKL1196M 4.9 HDAC1 10,800 HDAC6 1030 HDAC8 1690 HDAC11 16,600	A549 0.33 ± 0.03 (48 h) HepG2 0.59 ± 0.09 MCF7 0.55 ± 0.01 U87MG 0.62 ± 0.10 H2228 0.44 ± 0.06	[54]
**10**	ALKL1196M 34.28 ALKG1202R 2.74 ALKF1174L 9.23 HDAC1 240 ± 20 HDAC7 > 10,000 HDAC6 > 10,000 HDAC11 > 10,000	H2228 4.81 ± 0.90 MCF-7 5.30 ± 1.81 A549 9.72 ± 5.74 SK-N-BE2 0.69 ± 0.15	[56]
Brigatinib	ALKL1196M 3.63 ALKG1202R 14.58 ALKF1174L 1.04	H2228 2.01 ± 1.59 MCF-7 4.78 ± 1.14 A549 1.02 ± 0.35 SK-N-BE2 3.24 ± 1.53	
SAHA	–	H2228 7.26 ± 1.28 MCF-7 8.56 ± 4.48 A549_T_ 1.55 ± 0.37 SK-N-BE2 1.29 ± 0.04	

To evaluate the correlation between in vitro effects and tumor growth inhibition in vivo, compound **9** was used to treat A549 adenocarcinoma xenografts in immunodeficient BALB/c mice. At doses of 10 mg/kg and 20 mg/kg, the average tumor volume on day 21 of treatment decreased by 68% and 85%, respectively. The inhibitory effect was maintained 10 days after the cessation of therapy, with 80% and 86% inhibition on day 31.

The dual ALK/HDAC inhibitor **9** exhibited weak activity against CYP450 enzymes responsible for oxidation and metabolism, unlike selective ALK and HDAC inhibitors. Therefore, low CYP450 inhibition minimizes the risk of drug accumulation and side effects, confirming the compound’s safety for further studies.

In a 2025 study, Kong et al. [56] proposed compound **10** (Figure 3) as a dual ALK/HDAC inhibitor. It contains an o-aminobenzamide moiety as the ZBG and brigatinib as the ALK inhibitor moiety. The aminopyrimidine moiety formed two key hydrogen bonds with the MET-1199 residue in ALK. Molecular docking suggested that compound **10** could enter the active site of HDAC2 via the benzamide moiety chelating the catalytic zinc ion and forming a hydrogen bond with GLY-154.

Antiproliferative activity was evaluated using ALK-dependent H2228 (EML4-ALK) tumor cells. The activity observed was superior to that of SAHA (Table 2), but lower than that of brigatinib. Assessment of MCF-7 (breast cancer), A549 (non-small cell lung cancer), and SK-N-BE2 (neuroblastoma) cell lines showed stronger activity only against SK-N-BE2 cells compared to brigatinib.

Additionally, the compound demonstrated selectivity for the HDAC1 isoform and greater activity against the ALKG1202R mutant compared to brigatinib.

### 2.3. Dual AXL/HDAC Inhibitors

AXL proteins are part of the TAM family of receptor tyrosine kinases, which include Tyro3 and Mer [57]. Increased expression of AXL is associated with several types of human tumors, including liver cancer, lung cancer, breast cancer, stomach cancer, colorectal cancer, and prostate cancer [58,59,60]. Abnormal activation of the AXL signaling pathway leads to epithelial–mesenchymal transition in tumor cells, increasing their migration and invasiveness [61]. Gas6 is a soluble glycoprotein that binds to TAM receptors, including AXL [62]. It has the highest affinity for AXL and promotes its activation, which promotes cell survival and proliferation [63]. The Gas6/AXL pathway is also involved in cell invasion, migration, and angiogenesis [63].

**Figure 4:**
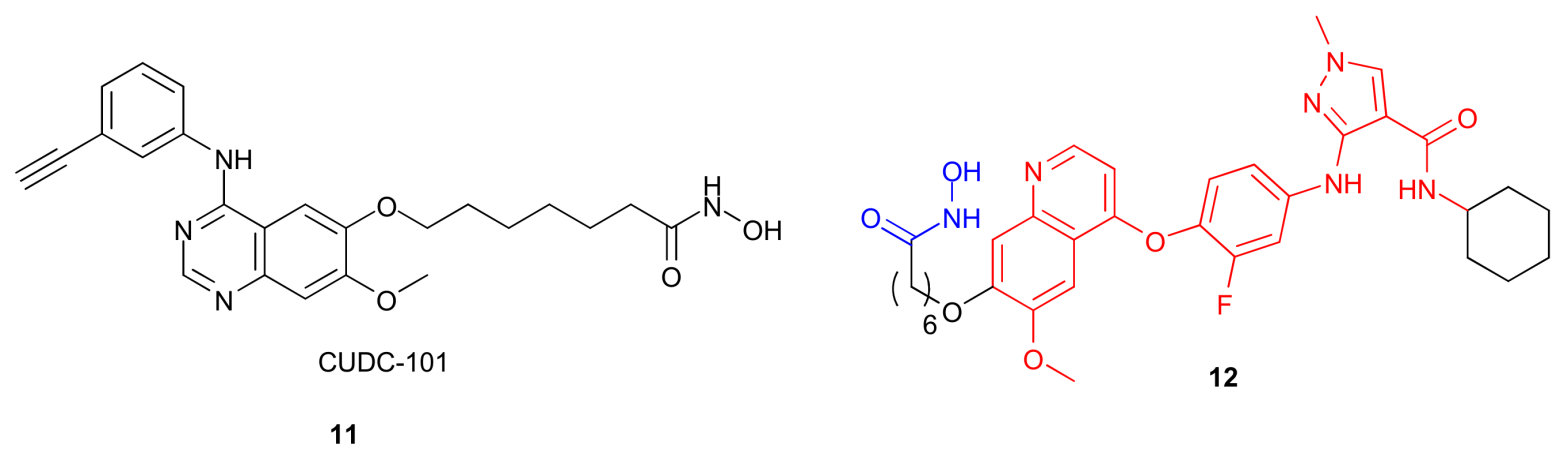
Structures of dual AXL/HDAC inhibitors. The fragment of the molecule responsible for AXL inhibition is marked in red, and HDAC inhibition is marked in blue.

HDACs with high affinity for HDAC2 induce apoptosis and cell cycle arrest by suppressing the expression of AXL and related molecules induced by mitomycin C (MMC) [64]. The multitargeted HDAC, EGFR, and HER2 inhibitor CUDC-101 (compound **11**, Figure 4) has been reported to downregulate AXL levels to overcome treatment resistance caused by AXL overexpression [65].

The clinical application of small-molecule AXL inhibitors such as TP0903, BGB324 (also known as bemcentinib), BMS-777607 and NPS-1034 is significantly limited due to rapid resistance development when used in monotherapy or radiotherapy for tumors [66,67].

Qiao et al. [68] investigated the antitumor activity of Hit-3, a dual AXL/HDAC inhibitor (compound **12**, Figure 4), in vitro and in vivo. The compound was developed based on known inhibitors of AXL (NPS-1034) and HDAC (SAHA). In vitro studies demonstrated potent cytotoxic activity against various cancer cell lines, including HCT116 colorectal, A549 lung adenocarcinoma, MCF-7 breast, HepG2 liver, HeLa cervical, H1975 EGFRi-resistant lung, and H460-resistant lung cancer cells (Table 3).

The inhibitory effect of Hit-3 is most pronounced against the HDAC2 isoform compared to HDAC1 and HDAC3–11, indicating selectivity. Its potency is greater than that of positive controls (AXL TP0903 and HDAC SAHA inhibitors), with IC_50_ values exceeding those by 2.8-fold and 2.2-fold, respectively (Table 3).

An in vivo analysis of Hit-3’s antitumor activity was performed in BALB/c mice that were transplanted with colorectal cancer cells (HCT116). Compared to the control group, which had a tumor volume of approximately 2500 mm^3^ after 12 days, administration of the substance at doses of 1, 10, and 20 milligrams per kilogram resulted in a significant reduction in tumor volume. The tumor volumes were approximately 1000, 500, and 5 mm^3^, respectively. Animal body weight remained stable, confirming the efficacy and safety of Hit-3.

### 2.4. Dual HER2/HDAC Inhibitors

HER2 is a member of the human epidermal growth factor receptor (EGFR) tyrosine kinase family [69]. Overexpression of this receptor is associated with the development of various types of tumors [70], particularly breast and gastric cancers [71].

Among HER2 inhibitors, lapatinib (GW 572016) has shown significant clinical activity in patients with HER2-positive advanced breast cancer (Phase II clinical trials) [72]. However, lapatinib is only effective when used in combination with capecitabine for patients with breast cancer [73]. Advancing research on dual-target HER2/HDAC1 inhibitors could lead to more effective and personalized treatments for HER2-positive breast cancer than combination therapy.

**Figure 5:**
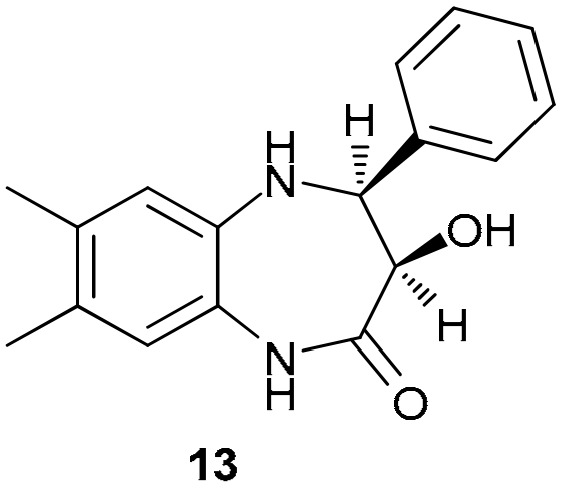
Structure of a dual HER2/HDAC inhibitor.

In one study [74], a benzodiazepine compound (compound **13**, Figure 5) was examined as a dual HER2/HDAC inhibitor. The presence of 3-hydroxy, 2H-1,5-benzodiazepin-2-one in the structure of this compound is predicted to coordinate zinc ions of HDAC. The same authors in an earlier study [75] demonstrated that this compound complied with Lipinski’s rule of five and exhibited good oral bioavailability and low toxicity in an in silico study. Molecular docking demonstrated that compound **13** formed hydrogen bonds with Thr798, Thr862, and Ser78 on HER2, as well as stable hydrophobic contacts with Lys753, Val734, Ala751, Leu785, Phe864, Leu755, and Leu796. In HDAC1, it was coordinated by Zn^2+^ ions and residues Gly149, Tyr303, Asp176, and His140, with additional stabilization due to hydrophobic interactions with Met30, Leu139, Phe150, Cys151, His178, Phe205 and Gly301 [74].

Compound **13** effectively inhibits HER2 and HDAC1 in the nanomolar range, similar to lapatinib and trichostatin A. It shows a pronounced antitumor effect against HCT-116, HepG-2, and MCF-7 cell lines (Table 4), with reduced toxicity to WI-38 fibroblasts, indicating selectivity. In HepG-2 cells, the compound induces apoptosis: The expression of caspase 3 and BAX increases eightfold, while Bcl-2 decreases. The proportion of early (15.14%) and late (24.72%) apoptosis, as well as necrosis (3.72%), significantly exceeds control values (1.42%).

### 2.5. Dual VEGFR/HDAC Inhibitors

VEGFs transmit signals through receptor tyrosine kinases on the cell surface. Vascular endothelial growth factor receptor kinase 2 (VEGFR-2), also known as transmembrane receptor kinase type III, is a key signaling receptor involved in angiogenesis [76]. VEGFR-2 is expressed on both vascular endothelial cells and lymphatic endothelial cells, and its inhibition can lead to an effective anti-tumor angiogenic response. Monoclonal antibodies and small-molecule inhibitors targeting VEGFR-2 are approved for the treatment of solid tumors, including ramucirumab, sorafenib, apatinib, vandetanib, pazopanib, cabozantinib, and fruquintinib [77]. Fruquintinib, approved in 2018 for the treatment of metastatic colorectal cancer in China, has shown promising results, but drug resistance has developed in most patients receiving VEGFR inhibitors, limiting their efficacy to 10–12% [78,79,80].

The combination of histone deacetylase inhibitors (HDAC inhibitors) with VEGFR-2 inhibitors holds great promise for overcoming resistance to VEGFR-2 inhibition and enhancing antitumor activity. Several dual HDAC/VEGFR-2 inhibitors have been developed, including pazopanib-based [81] and vandetanib-based inhibitors [82,83].

**Figure 6:**
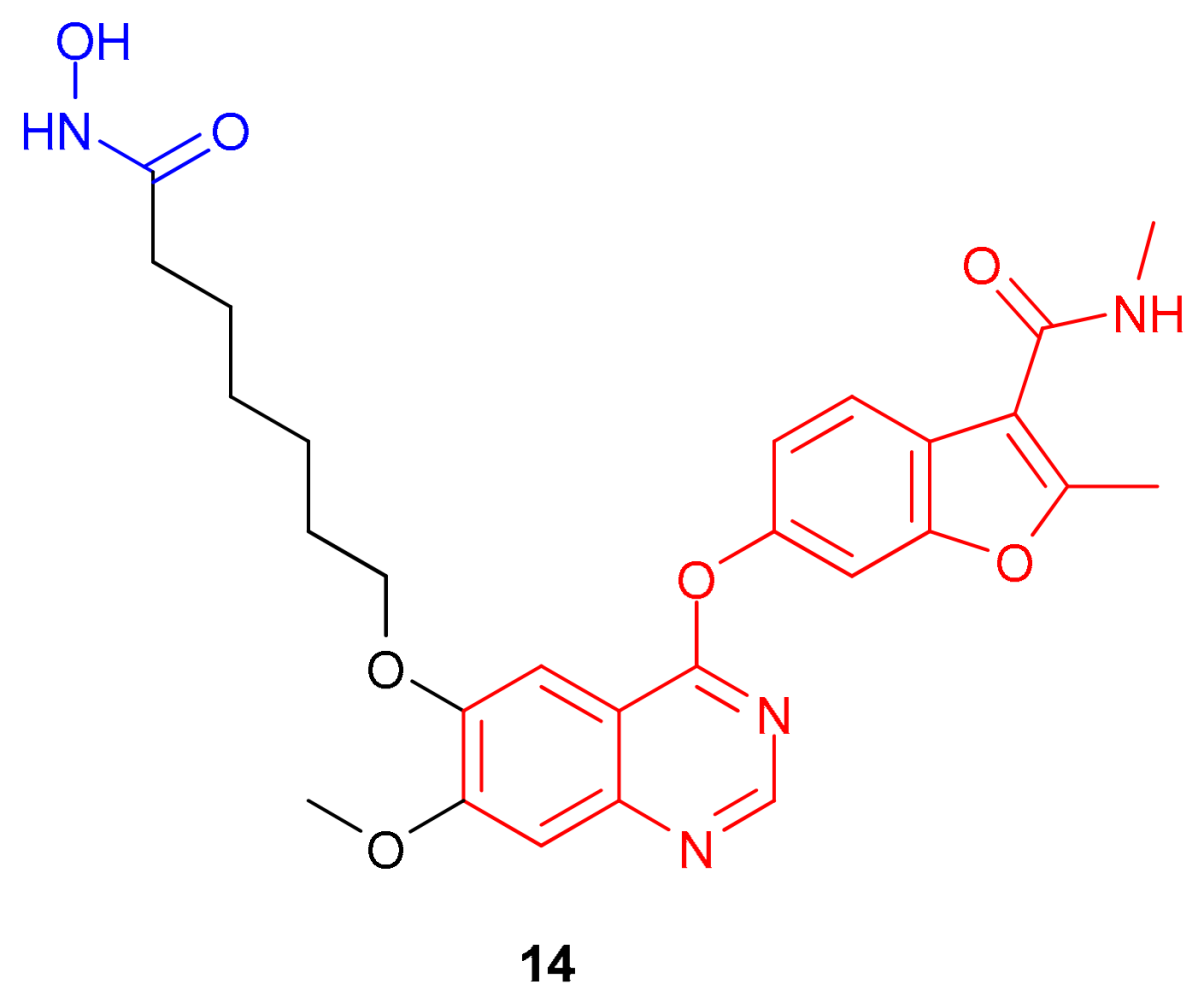
Structure of a dual VEGFR/HDAC inhibitor. The fragment of the molecule responsible for VEGFR inhibition is marked in red, and HDAC inhibition is marked in blue.

In 2023, Gao et al. [84] synthesized a dual VEGFR-2/HDAC inhibitor, 4-(benzofuran-6-yloxy)quinazoline (compound **14**, Figure 6), using the pharmacophores of the VEGFR-2 inhibitor fruquintinib and the HDAC inhibitor SAHA to model the new compound. The authors retained the quinazoline and benzofuran moieties of fruquintinib to optimally combine the HDACi and fruquintinib pharmacophores. These moieties mediate key interactions with VEGFR-2, including hydrogen bonds between the quinazoline ring nitrogen atoms and Cys919 in the hinge region and THR916, as well as hydrophobic interactions in the hydrophobic ATP-binding pocket. They also added a linker and the ZBG of histone deacetylase inhibitors at position 6 of the quinazoline ring. According to molecular docking data, the hydroxamic acid tail of compound **14** forms additional hydrogen bonds with Asp1033 and Arg1032 in the solvent region of VEGFR-2. Molecular docking also revealed that the 4-(benzofuran-6-yloxy)quinazoline molecule was located at the entrance to the active site of HDAC1 and that the methoxy group on the quinazoline ring and the oxygen atom from benzofuran formed hydrogen bonds with amino acids Phe198 and Tyr91, respectively.

The authors explain their choice of an aliphatic linker and a hydroxamic acid moiety over an aromatic linker and *o*-phenylenediamine as the ZBGs by their greater effectiveness in inhibiting HDAC1.

Inhibitory activity studies demonstrated inhibition of VEGFR-2 and HDAC1 in the nanomolar concentration range. In vitro cytotoxicity assays demonstrated good activity against MCF-7 breast cancer cells, HeLa human cervical cancer cells, and A549 human lung cancer cells, with IC_50_ values comparable to those of the SAHA and fruquintinib combination (Table 5). Significantly higher activity against A549 lung cancer cells has been observed compared to the SAHA and fruquintinib combination.

To investigate the cellular mechanisms of action of compound **14**, we tested its effects on apoptosis and the cell cycle in HeLa cells using flow cytometry. The apoptosis assay showed a significant dose-dependent increase in the number of apoptotic cells (7.69%, 18.26%, and 24.49% at concentrations of 0.75 μM, 1.5 μM, and 3 μM, respectively).

Compound **14** also exhibited inhibitory activity against human umbilical vein endothelial cells, suppressing the formation of tubular structures and confirming its antiangiogenic effect.

### 2.6. Dual FLT3/HDAC Inhibitors

The follicle tyrosine kinase 3 (FLT3) gene is a member of the receptor tyrosine kinase family. When the FLT3 ligand binds to its extracellular domain, it activates signaling pathways such as MAPK and PI3K/protein kinase B. These pathways are involved in the survival, maturation, and proliferation of hematopoietic cells [85,86]. These processes are essential for the development of the immune system. FLT3 can have both proliferative and anti-apoptotic effects on myeloid and lymphoid cells. These effects often occur in combination with other cytokines [87]. It is overexpressed in many cases of acute leukemia [88].

First-generation FLT3 inhibitors, such as sunitinib, sorafenib, and midostaurin, are not specific to the FLT3 kinase, but they also inhibit other kinases such as KIT, PDGFR, VEGFR, RAS/RAF, and JAK2 [89]. These inhibitors are effective against a range of targets and can cause side effects. Second-generation inhibitors like quizartinib, crenolanib, gilteritinib, and tandutinib have improved specificity and reduced toxicity. These newer drugs have been shown to be more effective at targeting the FLT3 kinase specifically. Midostaurin and gilteritinib have been approved for use in the USA as FLT3 inhibitors [89].

However, despite the proven clinical efficacy of these drugs, monotherapy with FLT3 inhibitors is often limited by the rapid development of acquired resistance, leading to transient therapeutic responses and unacceptably high relapse rates in patients with FLT3-mediated hematological malignancies.

Current studies have linked resistance to FLT3 inhibitors with epigenetic remodeling. Increased HDAC8 has been shown to confer resistance to quizartinib in MV4-11 and MOLM13 cells (FLT3-ITD+ AML). Furthermore, the expression of HDAC1, 2, 3, and 6 was significantly elevated in quizartinib-resistant MOLM13 clones compared to parental cells [90,91]. These findings provide a compelling rationale for the development of dual-targeting agents that target FLT3 and HDACs.

**Figure 7:**
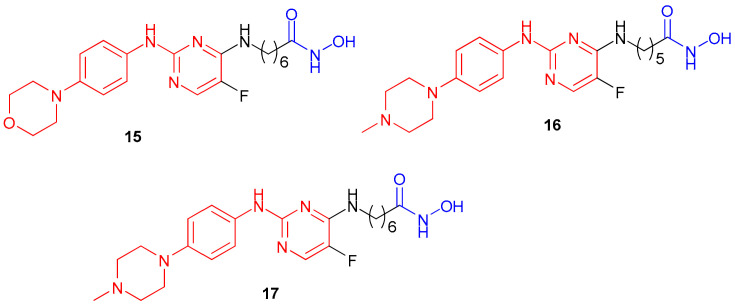
Structures of dual FLT3/HDAC inhibitors. The fragment of the molecule responsible for FLT3 inhibition is marked in red, and HDAC inhibition is marked in blue.

The authors of the study [92] synthesized compounds **15**–**17** (Figure 7), which contain an aminopyrimidine core (the pharmacophore for FLT3) and a hydroxamic acid as a ZBG. The aminopyrimidine binds to FLT3 through a hydrogen bond with the carbonyl oxygen of Leu616, and the morpholinophenyl group occupies the hydrophobic back pocket through H-π interactions with Phe691 and Val624.

Compounds **15**–**17** showed potent inhibitory activity against HDAC1 (Table 6). Derivatives **15** and **17**, with long hydrophobic linkers, provided optimal inhibition in the nanomolar range. Moreover, they exhibited selective dual-target activity. HDAC1 inhibition was 1.2–4.6 times more potent than SAHA, while FLT3 inhibition was 44–150 times more effective than tandutinib.

Compound **17** showed selectivity for HDAC1 and HDAC6 isoforms over HDAC4 and HDAC8, which are involved in oncogenesis and cancer progression. It also showed strong inhibitory activity against the mutant FLT3D835Y.

Furthermore, the synthesized compounds had a potent effect on hematological malignancy cells, including MV-4-11, Molt4, K562, and Jeko-1. Compound **17** demonstrated selectivity towards tumor cells compared to normal HaCaT cells.

Compared to SAHA, compound **17** inhibited FLT3 and its downstream signaling molecule p-STAT5 in MV-4-11 cells, blocking FLT3 activation and increasing acetylated histone H3 levels, indicating suppression of HDAC activity. Western blotting revealed a concomitant increase in proapoptotic protein Bax levels in MV-4-11 cells treated with compound **17** compared to those treated with SAHA.

Metabolic stability studies showed that **17** had a half-life of 165 min and an intrinsic clearance rate of 7.56 mL/min/kg in human liver microsomes, indicating good metabolic stability. Further assessment of **17’s** pharmacokinetic parameters in a SD rat model showed an oral bioavailability of 19.5% and a half-life of 2.21 h, and intraperitoneal administration yielded a bioavailability of 49%.

An in vivo study in NOD/SCID mice bearing xenografted Jeko-1 cells showed that at doses of 15 and 30 mg/kg, tumor growth inhibition values were 37.14% and 53.34%, respectively. These values were higher than those observed in the SAHA group (21.79%). A hemolysis test showed no hemolysis at concentrations of 50, 250 nM, 1, and 10 μM.

**Figure 8:**
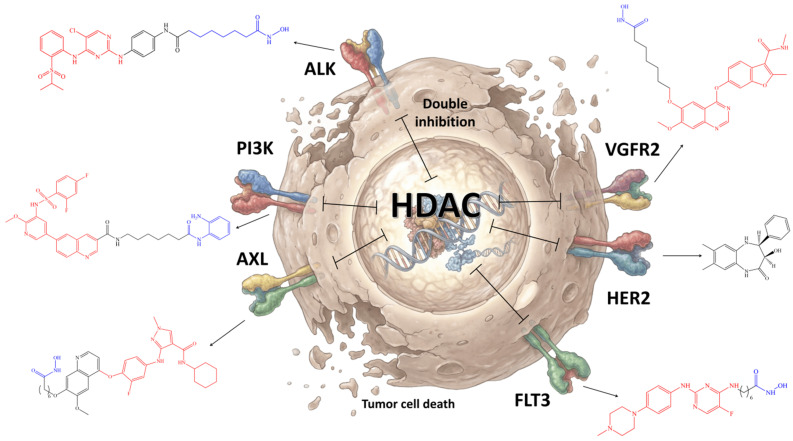
Examples of dual HDAC inhibitors and tumor cell membrane receptors. The fragment of the molecule responsible for tumor cell membrane receptors inhibition is marked in red, and HDAC inhibition is marked in blue.

Thus, dual inhibitors of cell membrane receptors (PI3K, ALK, AXL, HER2, VEGFR, and FLT3), as well as HDAC, demonstrate high antitumor potential through the synergistic inhibition of signaling pathways and epigenetic regulation (Figure 8). This overcomes resistance to monotherapy.

The synthesized compounds exhibit nanomolar activity against target enzymes and tumor cells in vitro, with selectivity for specific HDAC isoforms and certain tumor cells. Notable examples include CUDC-907 (**2**) for group 3 medulloblastoma, Hit-3 (**12**) for colorectal cancer, and compound **17** for FLT3-dependent leukemia. These compounds induce apoptosis, arrest the cell cycle (G0/G1 and G2/M phases), and suppress migration and angiogenesis. They also increase the levels of acetylated histones H3 and H4 and reduce phosphorylation of AKT, ERK, and FLT3. In an in vivo model, these compounds demonstrate tumor regression in mice, with a tumor growth inhibition (TGI) of up to 85%, without significant systemic toxicity.

## 3. Nuclear Targets

### 3.1. Dual Wee/HDAC Inhibitors

Wee1 is a nuclear protein kinase localized in the cell nucleus that plays a role in regulating the cell cycle. It works in cooperation with the p53 gene to control the transition between the G2/M and S phases of the cell cycle [93]. Wee1 helps to prevent cells with damaged DNA from entering mitosis, which can lead to mitotic catastrophe and apoptosis [94].

Wee1 inhibition has been identified as a promising approach for the treatment of acute myeloid leukemia (AML). In particular, the Wee1 inhibitor adavosertib (AZD1775), developed by AstraZeneca, is currently undergoing Phase II clinical trials as the first representative of this class [95]. However, compensatory activation of the cell cycle-regulating kinase CHK1 reduces the effectiveness of adavosertib in treating AML [96]. Because HDACs suppress the activation of CHK1 [97], combined inhibition of Wee1 and HDAC is expected to have a synergistic effect.

**Figure 9:**
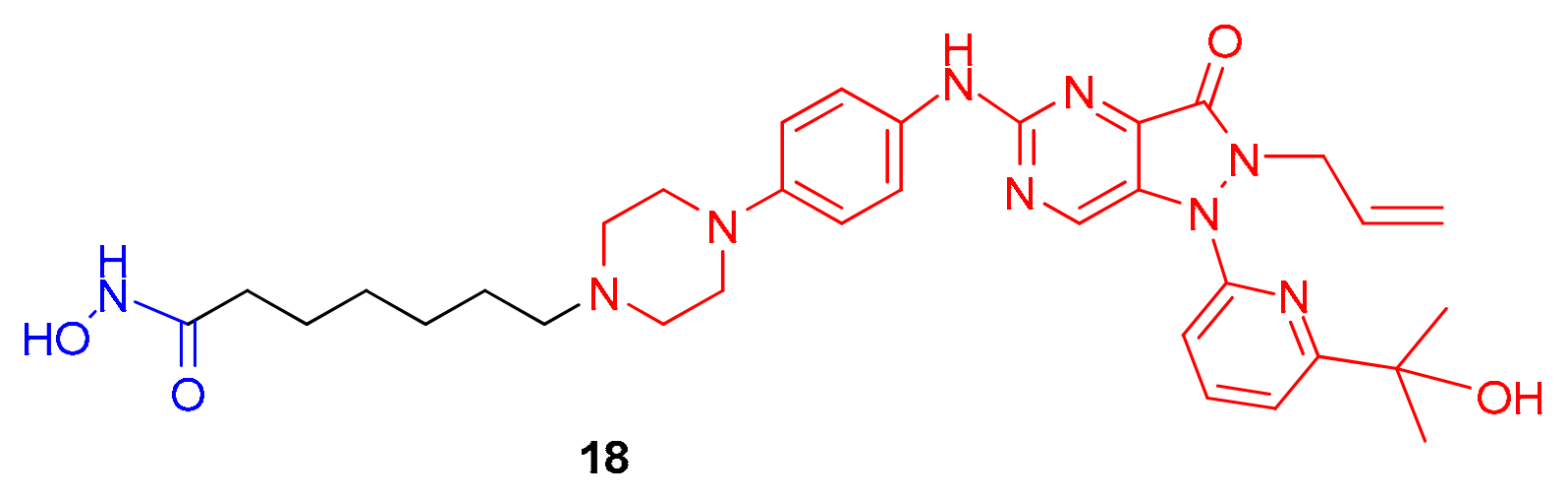
Structure of a dual Wee1/HDAC inhibitor. The fragment of the molecule responsible for Wee1 inhibition is marked in red, and HDAC inhibition is marked in blue.

The authors of the study [98] developed a novel dual Wee1/HDAC inhibitor, compound **18** (Figure 9). They used the pharmacophore fragments of adavosertib and SAHA to create this compound. Structural activity analysis revealed the following features: (1) the nitrogen atom of the amino group on the pyrimidine ring and the nitrogen of the pyrimidine interact with Cys379 in the hinge region; (2) the oxygen atom of pyrazolone forms hydrogen bonds with Asn376; (3) the hydroxyl group forms bonds with Asp463; and (4) the allyl group extends into a hydrophobic pocket, while the methylpiperazine resides in the solvent region. The authors explained the selection of methylpiperazine as the optimal site for HDACi pharmacophore insertion because there were no critical interactions in the binding pocket. Meanwhile, the SAHA structure underwent modification at the cap group.

The synthesized compound demonstrated inhibitory activity against Wee1 and HDAC1 in the nanomolar range, as well as excellent cytotoxicity towards the biphenotypic B-myelomonocytic leukemia cell line MV-4-11 (Table 7).

The pharmacokinetics of compound **18** in male SD rats, administered intraperitoneally at a dose of 10 mg/kg, showed rapid absorption with a peak concentration (C_max_) of 693.33 ± 91.09 ng/mL after a time of about 0.16 ± 0.14 h (t_max_). The bioavailability was high at 157.58%, and the half-life was 3.63 ± 1.14 h.

Intraperitoneal administration of three doses of 15, 30, and 60 mg/kg once daily for 21 days resulted in tumor growth inhibition (TGI) values of 52%, 70%, and 82%, respectively, which are superior to those observed with an oral dose of 30 mg/kg of AZD1775 (29% TGI). Additionally, no significant weight loss or mortality was observed during the treatment period in mice, indicating good tolerability of the drug. Western blotting of tumors confirmed the suppression of pCHK1 (**18** and combination vs. AZD1775) and enhancement of γH2AX with 60 mg/kg, supporting the synergistic mechanism of Wee1 and HDAC inhibition.

### 3.2. Dual DNMT/HDAC Inhibitors

Along with histone deacetylation, DNA methylation is another important epigenetic modification in cancer cells. DNA methylation plays a crucial role in regulating the expression of tumor suppressor genes, such as BRCA1 and RASSF1A in breast cancer and MGMT, MLH1 and WRN in colon cancer [99]. Aberrant activation of DNA methyltransferase (DNMT) enzymes leads to hypermethylation of CpG dinucleotides in CpG islands [100], which are normally unmethylated in healthy cells.

Among the approved DNMTIs (DNA methyltransferase inhibitors), nucleoside analogues such as azacitidine and decitabine have low specificity and require high doses, which are limited by their toxicity. Non-nucleoside DNMTIs have not been clinically approved, despite their notable potential [101].

It is known that the expression of DNMT1 and HDAC1 is significantly increased in breast cancer tissues compared to normal tissues [102]. In 2008, it was shown that HDACis can reduce the expression of DNMT1 protein inside the tumor cell nucleus, and inhibition of HDAC3 can increase the level of DNMT1 acetylation, thereby reducing its stability [103]. Therefore, the development of dual DNMT/HDAC inhibitors is a promising direction of research in the field of polypharmacology.

**Figure 10:**
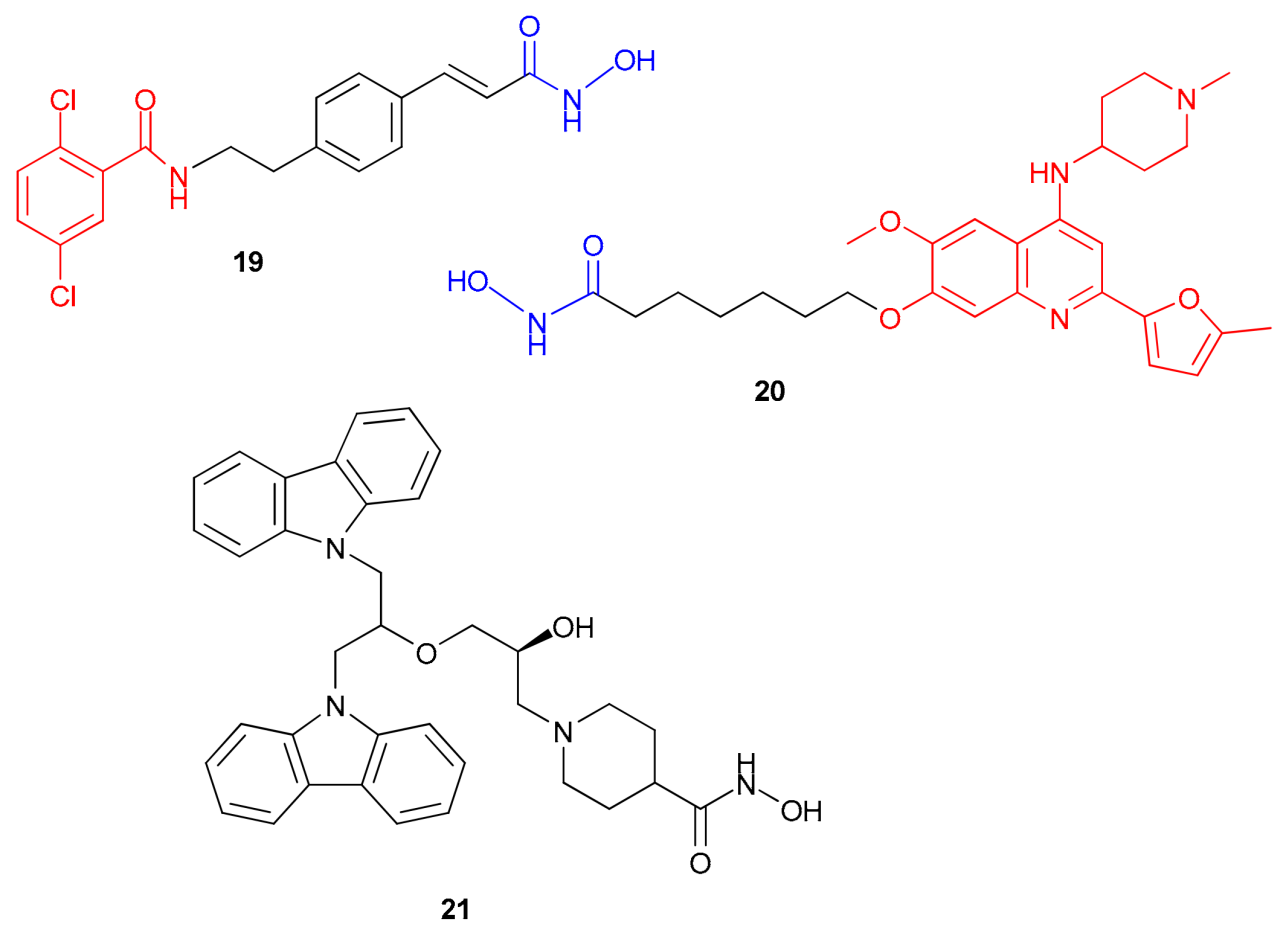
Structures of dual DNMT/HDAC inhibitors. The fragment of the molecule responsible for DNMT inhibition is marked in red, and HDAC inhibition is marked in blue.

The authors of [104] demonstrated that a dual inhibitor of DNMT1 and HDAC (compound **19**, Figure 10), derived from the DNMT inhibitor NSC-319745 and the HDAC inhibitor panobinostat [105], effectively suppressed the proliferation, migration, and invasion of the breast cancer cells MDA-MB-453 and BT-474 by inducing apoptosis.

The authors of [104] demonstrated that 24 h treatment with compound **19** increased histone H3 acetylation in MDA-MB-453 and BT-474 breast cancer cells. It also reduced DNMT1 expression and exhibited a moderate antiproliferative effect on MCF-7 cells and a more pronounced effect on other cell lines, as shown in Table 8. Additionally, **19** induced apoptosis and G2 cell cycle arrest in a concentration-dependent manner in vitro. In vivo studies showed that when administered at a dose of 50 mg/kg, **19** reduced tumor size by more than 30% in BALB/c mice compared to control groups. Furthermore, the compound reduced DNMT1 and HDAC1 levels, increased H3 acetylation levels, and enhanced caspase-3 and p27 expression. It also stimulated endogenous retroviral elements, reactivated tumor suppressor genes, increased immunogenicity, and induced the secretion of cytotoxic cytokines from macrophages, NK cells, and CD8 T cells, activating the RIG-I-MAVS pathway and enhancing type I/III interferon production. This led to increased ISG transcription and chemokine secretion. In MDA-MB-453 and BT-474 cells, MDA5 and RIG-I signaling were increased, as was JAK–STAT signaling. The combination of an anti-PD-L1 antibody and a DNMT/HDAC inhibitor produced an additive effect in vivo, confirming the immunomodulatory properties of this inhibitor and its synergy with immune checkpoint blockade.

In 2025 [106], the same scientific group synthesized a dual DNMT/HDAC inhibitor (compound **20**, Figure 10), which demonstrated potent inhibitory activity against DNMT1 and HDAC1 and selectivity for DNMT1 over DNMT3A and DNMT3B. It also had good antitumor potential against triple-negative breast cancer cell lines MDA-MB-231, MDA-MB-468, MDA-MB-453, MDA-MB-157, HCC1937, HCC1143, HCC38, and BT-549, and the non-triple-negative (T-47D) breast cancer subtype (Table 8).

This compound, which contains a linker at position 7 of the quinoline ring, was designed based on the DNA methyltransferase 1 (DNMT1) inhibitor CM-272 and SAHA. Molecular docking showed deep penetration of the hydroxamic moiety Y7 into the active site of DNMT1, forming hydrogen bonds with Leu1154 and Phe1148. The 2-methylfuran and aliphatic linkers were also located in a hydrophobic pocket, interacting with Met1172 to increase affinity for DNMT1 compared to the substituent on the nitrogen atom of the piperidine ring. In vivo pharmacokinetic studies in rats after intravenous administration of compound **20** at 1 mg/kg revealed a moderate half-life (T_1/2_ = 1.67 h), a high volume of distribution (V_ss_ = 109.53 L/kg), and a high clearance (Cl = 1207.46 milliliters/minute/kg), indicating good tissue distribution and rapid elimination. In vivo experiments showed that compound **20**, at doses of 5 and 15 mg/kg, significantly suppressed tumor growth in both xenograft and transgenic breast cancer models. This compound outperformed the decitabine–SAHA combination without causing significant toxicity. Histological analysis of lung tissue revealed pronounced metastases in the control group, but a significant decrease in the number of pulmonary nodules was observed in the compound **20** and decitabine–SAHA treatment groups. The most pronounced antimetastatic effect was seen in the group receiving compound **20** at a dose of 15 mg/kg.

Furthermore, the use of DNMT/HDAC inhibitors is not only effective in terms of antiproliferative action but also increases the expression of interferon receptors (IFN), enhancing the effects of immune checkpoint therapy. The dual DNMT/HDAC inhibitor **21** exhibited antitumor activity in LS 174T, SW480, and Caco 2 cell lines (Table 8) and suppressed tumor growth in immunocompetent mice [107]. Compound **21** induced ERVs and increased intracellular dsRNA levels, which activated the RIG I/MDA5–MAVS signaling pathway and IFN expression. Moreover, it increased DC and T cell infiltration and enhanced the efficacy of anti-PD L1 therapy in MC38 and CT26 models.

Treatment of LS 174 T cells with compound **21** at concentrations ranging from 2 to 16 μM resulted in a dose-dependent inhibition of DNMT1 activity and an increase in H3K27 acetylation. Additionally, there was a significant increase in the levels of p-STAT1 and ISGs (STAT1 and MDA5), indicating activation of the IFN signaling pathway.

Thus, combined inhibition of DNMT and HDAC synergistically enhances immunotherapy. However, compound **21** was less effective than SAHA and DC 517 against HCT 116, HT 29, DLD 1, LoVo, SW620, CT26, and MC38 cells, and the authors do not explain the reason for this decrease in activity.

Therefore, the development of small-molecule agents that can target both DNMTs and HDACs simultaneously is a promising strategy for breast cancer treatment.

### 3.3. Dual CDC25/HDAC Inhibitors

CDC25 is a protein that plays a role in the cell cycle. It is a phosphatase that removes phosphate groups from certain amino acids in proteins called cyclin-dependent kinases (CDKs). This process activates the CDKs, which in turn help the cell progress through the cell cycle. Dysregulation of CDC25 can lead to abnormal cell division and can be linked to various types of cancer. Therefore, it is an interesting target for research and cancer treatment [108].

Naphthoquinone derivatives, such as NSC95397, UPD-140, and Cpd-5, have been shown to influence CDC25 activity and cell cycle progression. These compounds irreversibly oxidize the cysteine residue in CDC25’s catalytic domain [109,110].

HDAC inhibitors reactivate tumor suppressor genes, such as p21, in tumor cells. This leads to the suppression of CDK/cyclin complexes, resulting in cell cycle arrest and differentiation. Conversely, CDC25 inhibitors also inhibit CDK activation, causing cell cycle arrest and apoptosis in cancer cells [111]. Therefore, simultaneous inhibition of both CDC25 and HDACs may produce a synergistic antiproliferative effect, arresting cancer cell growth.

**Figure 11:**
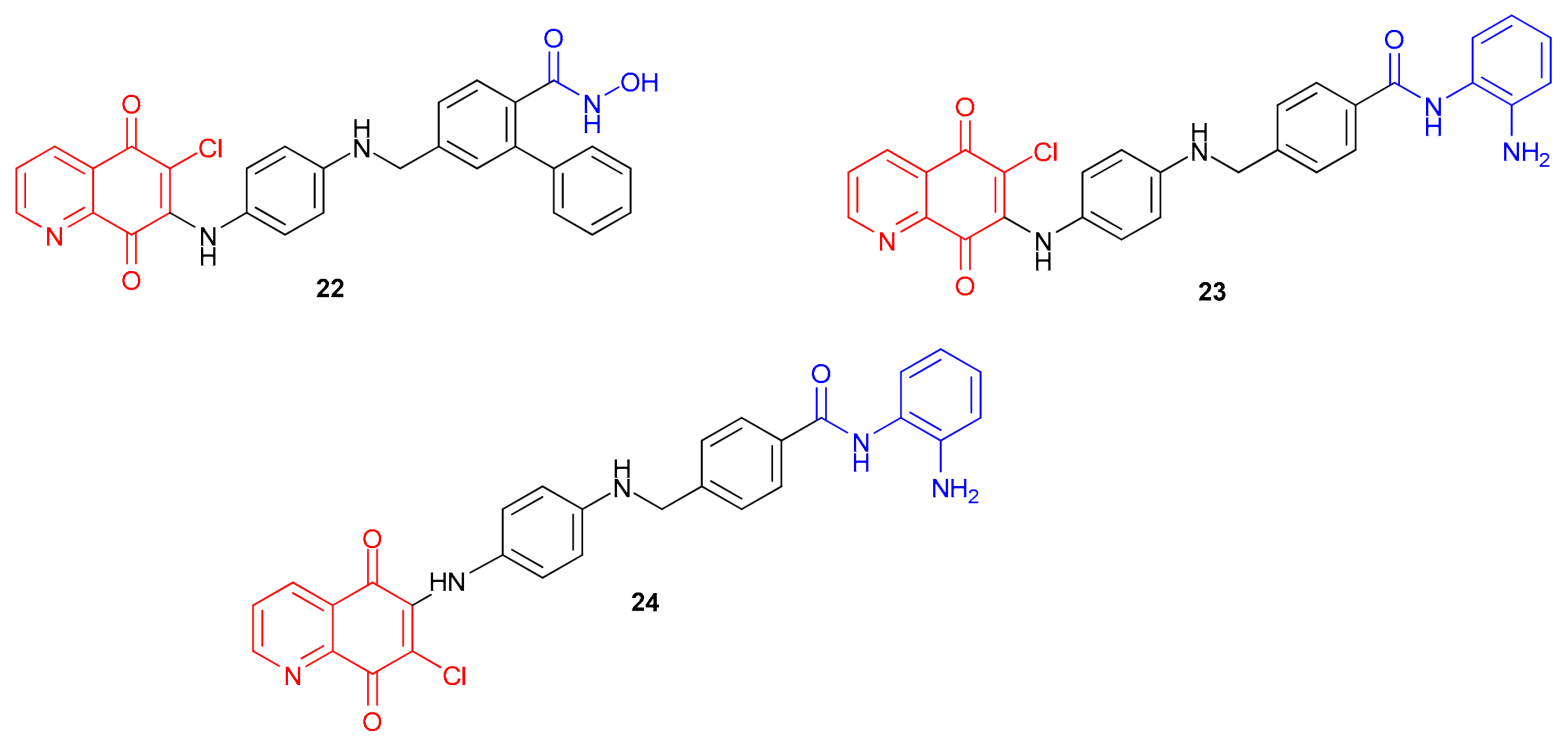
Structures of dual CDC25/HDAC inhibitors. The fragment of the molecule responsible for CDC25 inhibition is marked in red, and HDAC inhibition is marked in blue.

In [112], compounds **22**, **23**, and **24** (Figure 11) were designed. These compounds contain a quinoline-5,8-dione moiety acting as the cap group for the CDC25 inhibitor core and a 2-aminobenzamide or hydroxamate moiety acting as a zinc-binding group for HDAC inhibitors. The quinoline-5,8-dione group was chosen as a cap group because of its interactions with aromatic amino acid residues near the outer region on the surface of the enzyme active site.

The authors evaluated the cytotoxicity of compounds **22**, **23**, and **24** against the triple-negative breast cancer cell line MDA MB 231 after 48 h of treatment and found that all three compounds demonstrated significant activity (Table 9). Compound **23** showed the highest level of cytotoxicity, outperforming both derivatives **22** and **24** as well as the reference HDAC inhibitor MS 275 and the individual combination of CDC25 and HDAC inhibitors. This suggests that it may be effective at simultaneously targeting both CDC25 and HDAC within a single molecule.

Treatment of MDA-MB-231 cells with compound **23** resulted in increased levels of histone H3/H4 acetylation similar to those observed with MS-275. However, it did not significantly affect SMC3 or alpha tubulin acetylation, suggesting specificity for class I histone deacetylases (HDACs) without significant effects on HDACs 6 and 8. Furthermore, **23** showed more potent inhibition of HDAC1 compared to MS-275 while maintaining comparable activity against HDACs 2 and 3. This confirms its status as a potent and specific class I HDAC inhibitor, supporting its potential as a promising candidate for further research.

**Figure 12:**
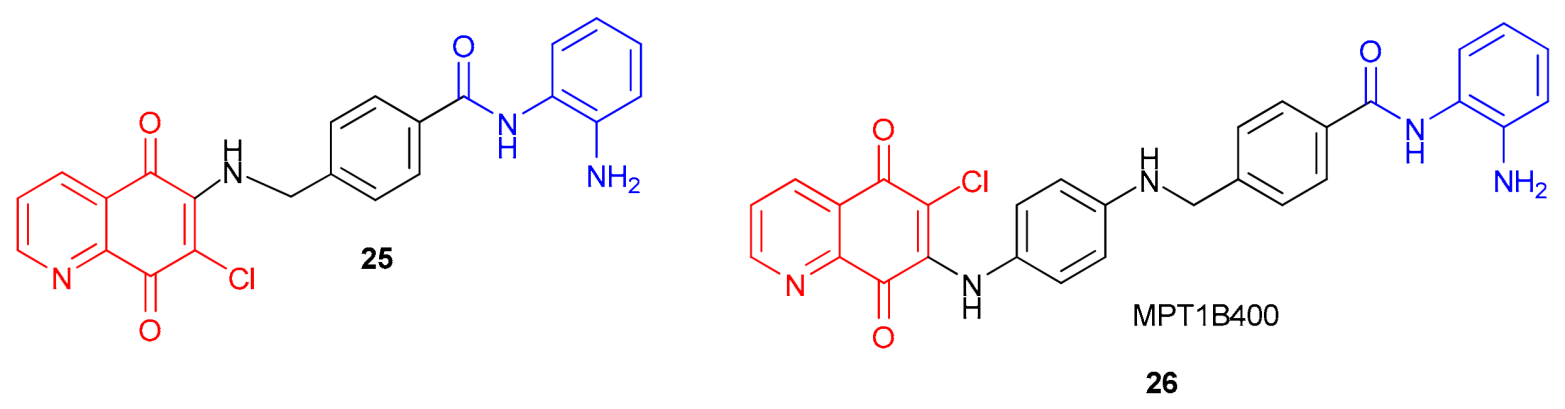
Structures of dual CDC25/HDAC inhibitors. The fragment of the molecule responsible for CDC25 inhibition is marked in red, and HDAC inhibition is marked in blue.

In work [113], the dual MPT1B394 inhibitor MPT1B400 (compound **25**, Figure 12) exhibited potent cytotoxicity against A172 glioblastoma cells (Table 9). Additionally, it showed favorable in vivo safety and survival results in an orthotopic GBM mouse model.

To assess the safety profile of compounds **25** and **26**, they were administered intraperitoneally to mice at a dose of 10 mg/kg, three times a week for two weeks. Body weight was monitored as an indicator of systemic toxicity. Compound **25** was found to be well tolerated with minimal weight loss. Cytotoxicity tests on parental (U-87MG, A172, P3) and TMZ-resistant (U-87MG-R, A172-R, P3-R) glioblastoma cell lines showed effective growth inhibition. This indicates the ability of compounds **25** and **26** to overcome chemoresistance in these cell lines.

To evaluate the therapeutic potential of the treatment in vivo, an orthotopic glioblastoma model was generated in C57BL/6 mice by stereotactically implanting CT-2A cells expressing luciferase into the right frontal lobe. Starting on day 6 after implantation, animals received treatment with **25** or **26** (5 mg/kg, intraperitoneally, three times a week until day 30). IVIS imaging showed a significant reduction in photon emission and stable suppression of intracranial GBM growth in the 25-treated group compared with the control (DMSO).

### 3.4. Dual CDK9/HDAC Inhibitors

Cyclin-dependent kinase 9 (CDK9) is a member of the serine/threonine protein kinase family [114] that controls the cell cycle. The CDK9–cyclin T1 complex forms the core component of the positive transcription elongation factor b (P-TEFb) complex [115]. P-TEFb plays a role in mRNA processing by phosphorylating the serine residue Ser2 of the carboxyl-terminal domain (CTD) of RNA polymerase II (RNAPII), precisely regulating RNA transcription elongation [116], and by promoting the action of the elongation factor MYC. Dysfunction of the regulated CDK9 signaling system and aberrant activation of the oncoprotein MYC have been identified, in particular, in triple-negative breast cancer [117], hepatocellular carcinoma [118], and other tumor types.

Given the potential of CDK9 inhibition as a cancer treatment strategy, numerous CDK9 modulators with antitumor activity have been identified over the past decades. Non-selective CDK inhibitors, such as flavopiridol, CDKI-73, and LDC000067, as well as selective CDK9 inhibitors like atuveciclib, enitociclib, and SLS009, have shown efficacy in in vitro and preclinical in vivo models [119]. Some of them are recommended for clinical use. For example, flavopiridol (TP-1287) has been FDA-approved since 1994 for the treatment of acute myeloid leukemia and Ewing sarcoma. In 2023, TP-1287 received orphan drug designation [120]. In 2023, the highly selective CDK9 inhibitor SLS009 (formerly GFH009) received Fast Track designation for the treatment of relapsed/refractory AML and peripheral T-cell lymphoma [120].

**Figure 13:**
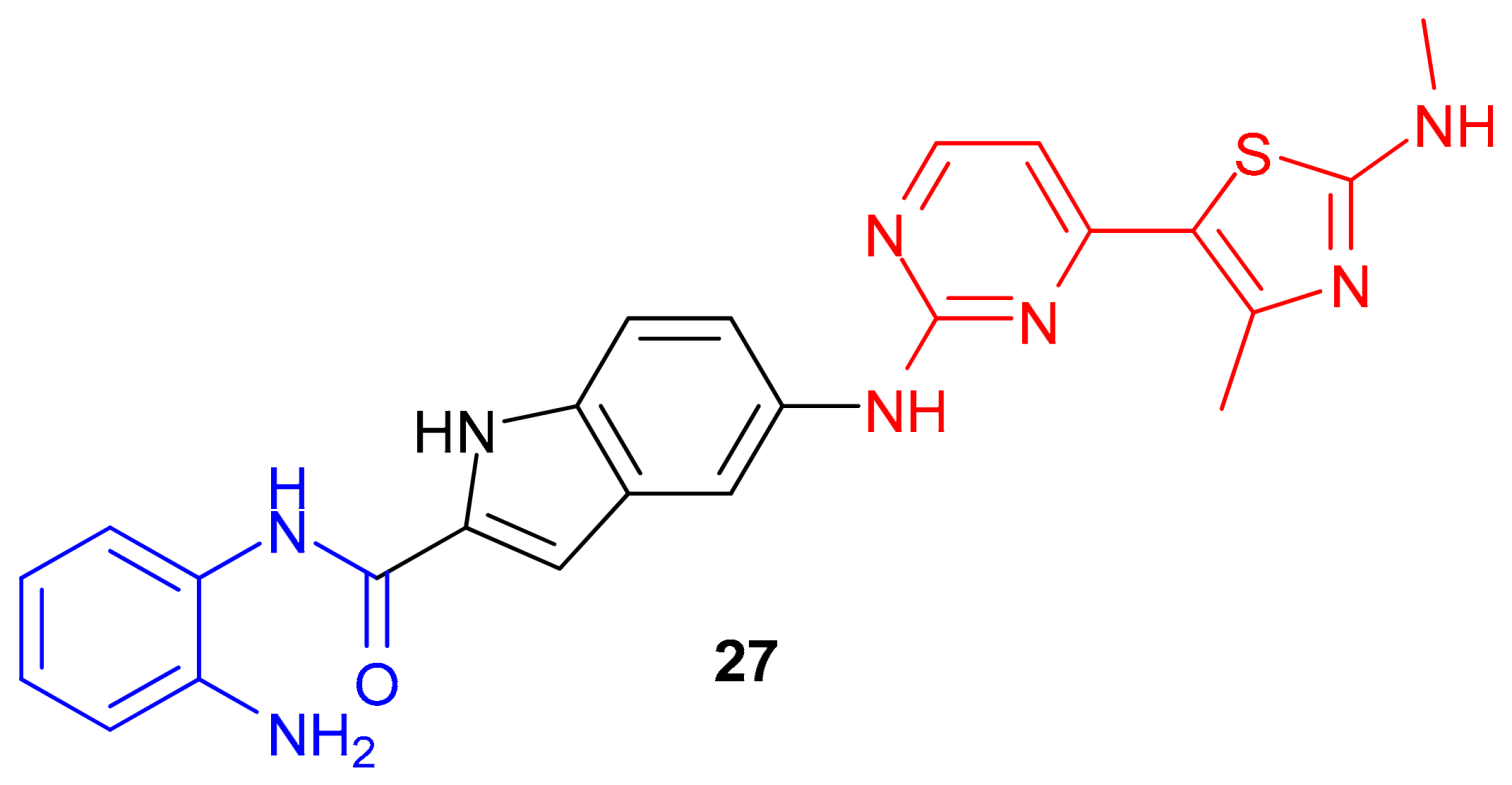
Structure of a dual CDK9/HDAC inhibitor. The fragment of the molecule responsible for CDK9 inhibition is marked in red, and HDAC inhibition is marked in blue.

Currently, dual CDK9/HDAC1 inhibitors are of interest. In particular, the dual-target CDK9/HDAC1 inhibitor **27** (Figure 13) was synthesized in [121] based on the class I HDAC inhibitor mocetinostat and the CDK9 inhibitor CDKI-73.

Molecular docking analysis revealed that the aminopyrimidine group of this molecule forms two hydrogen bonds with Cys106 in the hinge region of the protein. The *o*-aminoanilide group is oriented towards the solvent region of the enzyme and forms a hydrogen bond with the Asp109 residue. The 4-methyl-2-(methylamino)thiazol-5-yl group occupies a deep active pocket created by the Ala166, Asp167, Phe168, Gly169, Phe30, and Val33 residues.

Compound **27** exhibited pronounced antiproliferative activity against HeLa, MDA-MB-231, and HepG-2 cell lines (Table 10). It reduced the levels of CTD p-Ser2 of RNA polymerase II, Bcl-2, and Mcl-1, and increased H3 acetylation (Ac-H3), exceeding the effects of CDK9 (AZD-5438) and HDAC (mocetinostat) inhibitors. The IC_50_ against CDK9 was 170 nM, against HDAC1 was 1730 nM, and against HDAC3 was 1110 nM (Table 10). In MDA-MB-231 cells, compound **27** induced apoptosis and cell cycle arrest, outperforming the reference drugs.

In an in vivo MDA-MB-231 xenograft mouse model, TGI achieved 76.83% at 30 mg/kg after 10 days, with no signs of systemic toxicity (such as weight loss or organ damage).

Thus, compound **27** has potential as a candidate for the treatment of triple-negative breast cancer. However, further pharmacokinetic studies and a better understanding of its mechanism of action are needed.

### 3.5. Dual DYRK2/HDAC Inhibitors

DYRK2 (dual-specificity tyrosine phosphorylation-regulated kinase 2), a member of the class II DYRK family, possesses both tyrosine kinase and serine/threonine kinase activities [122]. DYRK2 has been shown to play a dual role in carcinogenesis: It acts as a tumor suppressor by phosphorylating p53 at Ser46, degrading c-JUN and c-MYC, and inhibiting SNAIL, but it also acts as an oncogene by activating the 26S proteasome through Rpt3 at Thr25 during the G1/S transition. Increased expression of DYRK2 is characteristic of several types of cancer, including prostate cancer [122], gastric adenocarcinoma [123], liposarcoma [124], multiple myeloma, and triple-negative breast cancer [125]. Inhibiting DYRK2 reduces proteasomal activity and cell proliferation [126]. However, currently available DYRK2 inhibitors are not sufficiently effective and selective [127], so there is a need to develop more potent and selective inhibitors for use in cancer treatment.

HDAC8 inhibition inhibits hepatocellular carcinoma proliferation by increasing p53 expression and acetylation of p53 at Lys382 [128]. A 2018 study demonstrated that a combination of the DYRK2 inhibitor imatinib and the HDAC8 inhibitor AR-42 is effective against chronic myeloid leukemia cells [129]. Therefore, dual targeting of DYRK2 and HDAC8 simultaneously may be an effective, safe, and promising strategy for tumor treatment.

**Figure 14:**
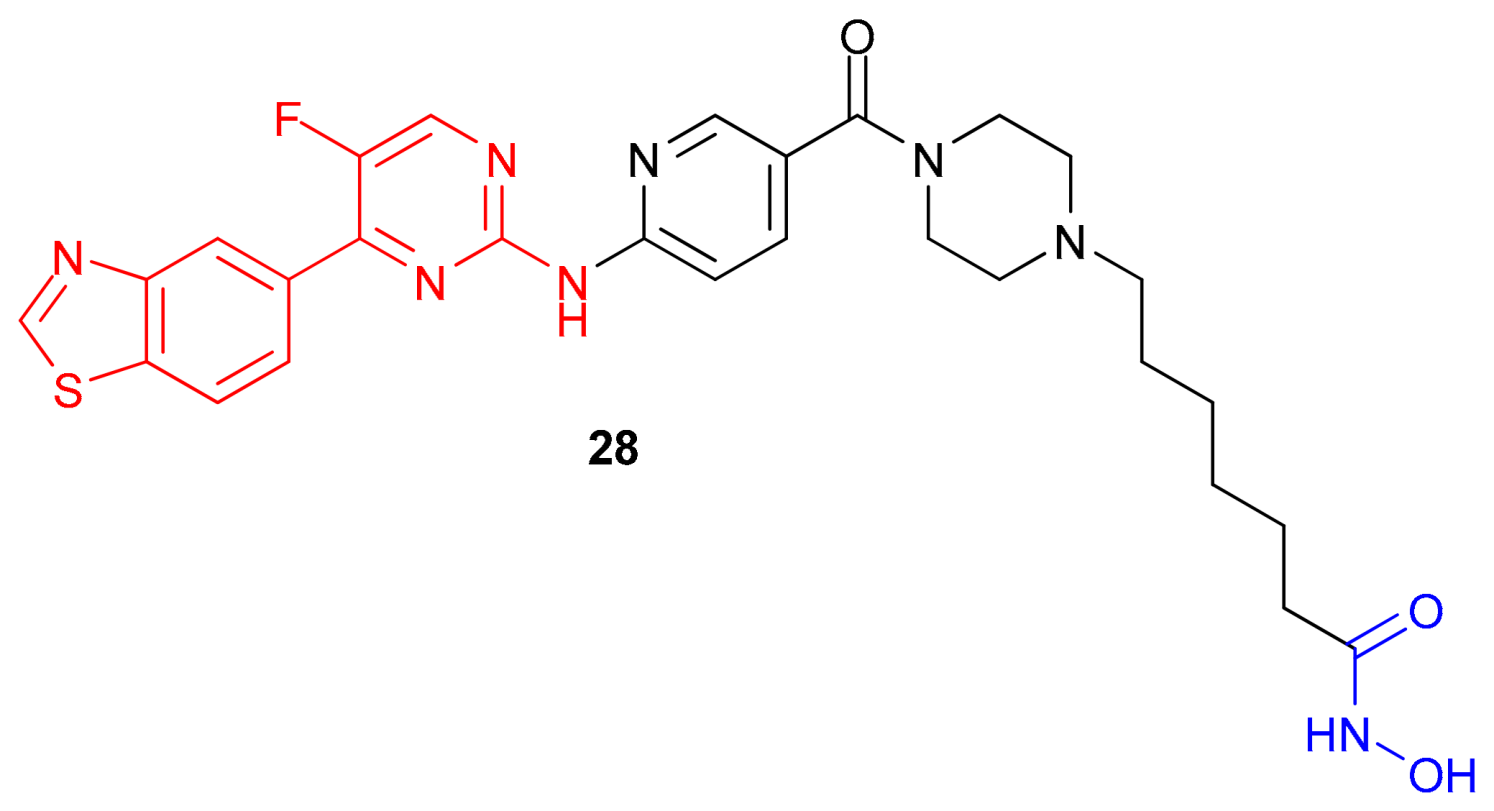
Structure of a dual DYRK2/HDAC inhibitor. The fragment of the molecule responsible for DYRK2 inhibition is marked in red, and HDAC inhibition is marked in blue.

In study [130], a dual DYRK2/HDAC8 inhibitor (compound **28**, Figure 14) was synthesized and tested for its antitumor activity in vitro and in vivo. The compound’s structure was based on the structures of the DYRK2 inhibitor YK-2-69 and the HDAC inhibitor SAHA.

The inhibitory activity study of compound **28** showed significant dual inhibition of both DYRK2 and HDAC8, with an IC_50_ value that was lower than those of the positive controls YK-2-69 and quisinostat (Table 11).

In vitro studies of compound **28**’s antiproliferative effects have shown good activity against three types of human liver cancer cells (SK-HEP-1, HuH-7, and HepG2), but low activity against normal hepatocytes L02. This indicates that compound **28** has selectivity. An in vivo study in a mouse xenograft model derived from SK-HEP-1 cells demonstrated a decrease in tumor volume and weight after administering compound **28** at a dose of 10 mg/kg for 24 days. Tumor volume decreased from 600 mm^3^ to approximately 100 mm^3^, and tumor weight decreased from approximately 0.7 g to 0.1 g, while mouse weight increased slowly, indicating safety. These data suggest that compound **28** may be a potent and safe DYRK2/HDAC8 inhibitor with potential for further preclinical and clinical research.

### 3.6. Dual BET/HDAC Inhibitors

Because epigenetic changes are reversible, proteins that interact with chromatin are promising targets for developing antitumor agents. Bromodomains (BRDs) recognize the ε-N-lysine acetylation of histones [131], coordinating chromatin complexes that contain BRD proteins, including the nuclear BRD4 protein, to promote carcinogenesis [132].

BET bromodomain inhibitors are currently being tested in clinical trials for various types of cancer [133]. OTX015 (birabresib), for example, has been tested in phase I trials for AML (acute myeloid leukemia), acute lymphoblastic leukemia (ALL), DLBC lymphoma, multiple myeloma, and solid tumors [134]. However, the potential use of these drugs is limited by their side effects, such as fatigue, hypertension, thrombocytopenia, and gastrointestinal bleeding.

In addition, current research is exploring the potential of combining BET and HDAC inhibitors to more effectively suppress oncogenic gene expression and tumor cell growth [134]. This is because HDACs play a significant role in regulating gene expression. For instance, a synergistic effect has been observed from the combined use of these inhibitors in various types of cancer, including pancreatic adenocarcinoma [135], Myc-induced lymphoma [136], bladder cancer [137], and melanoma [138]. However, using two drugs in combination therapy carries risks such as drug interactions, off-target toxicity, and additive effects.

**Figure 15:**
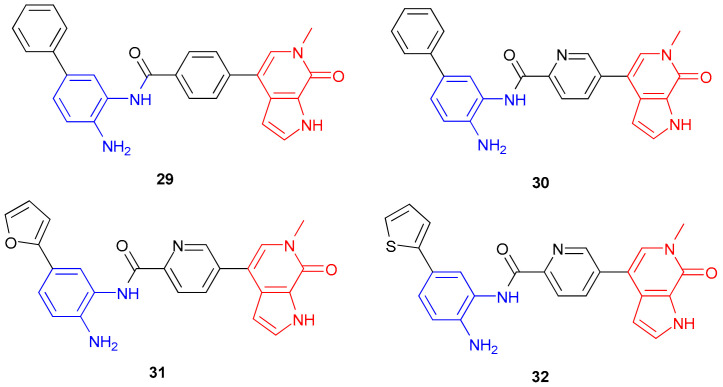
Structures of dual BRD4/HDAC inhibitors. The fragment of the molecule responsible for BRD4 inhibition is marked in red, and HDAC inhibition is marked in blue.

The authors of study [139] synthesized a series of dual-target BRD4/HDAC inhibitors (compounds **29**, **30**, **31**, and **32**), as shown in Figure 15. To design these molecules, they combined two pharmacophore scaffolds—the BET inhibitor MS436 and the HDAC1 inhibitor CI-944—with a benzamide group. In order to improve binding stability in the BRD4 bromodomain, they replaced the phenolic moiety of MS436 with a pyrrolopyridine moiety. This resulted in a threefold increase in binding affinity for compound **30** compared to the original phenyl group.

The resulting compounds showed improved inhibitory activity against HDAC1 and HDAC2 compared to CI-944 and MS-436 (Table 12). Compounds **29**, **30**, **31**, and **32** were selective for the HDAC1 and HDAC2 isoforms, with compound **32** exhibiting the best inhibitory activity among these compounds. It is worth noting that compound **32** contains a 2-thienyl group at position 5 on the aromatic ring.

A study of the anticancer activity against the pancreatic cancer cell line PaTu8988T showed lower IC_50_ values for compounds **29**–**32** compared to MS346 and CI-944, as well as their combination (Table 12). Analysis of histone H3 acetylation levels at K9/K14 residues showed the highest levels for compound **30**, which contains a phenyl group that binds to a narrow hydrophobic channel near the HDAC1 active site.

The authors also observed an increase in the expression of the tumor suppressor HEXIM1 and the proapoptotic protein p57, both of which are markers of BET inhibition. After treating the PaTu8988T cells with compounds **29**–**32** for 6 h at a concentration of 1 μM, all of the compounds increased the levels of HEXIM1 mRNA. Compound **29** had the most significant effect, while the levels of p57 mRNA were significantly increased by dual inhibitors **30** and **32** compared to MS436.

**Figure 16:**
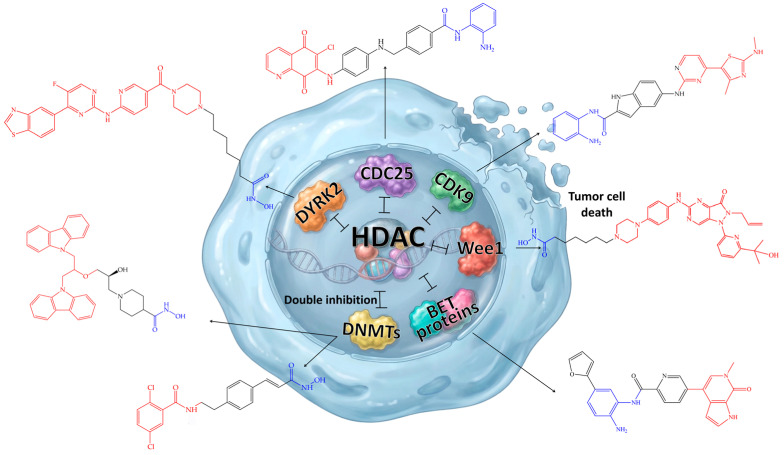
Examples of dual HDAC inhibitors and tumor cell nuclear molecular targets. The fragment of the molecule responsible for tumor cell nuclear molecular targets inhibition is marked in red, and HDAC inhibition is marked in blue.

Overall, dual inhibitors of HDACs and nuclear targets (Wee1, DNMT, CDC25, CDK9, DYRK2, and BET) (Figure 16) exhibit synergistic antiproliferative, apoptotic, and immunomodulatory effects in acute myeloid leukemia, breast cancer, glioblastoma, and hepatocellular carcinoma models.

Examples of these compounds—**18**, **19**, **20**, **23**, **27**, and **28**—outperform monofunctional reference compounds, such as adivosertib and SAHA, in vitro in terms of cytotoxicity (with an IC_50_ in the low nanomolar to submicromolar range) and tumor growth inhibition (up to 82%) in vivo. These compounds have a favorable pharmacokinetic profile and minimal toxicity, making them promising candidates for future polypharmacological therapies. However, further preclinical optimization is needed to overcome resistance and minimize side effects.

## 4. Cytotoxicity Studies Using 3D Models

Above, we mainly focused on 2D cultures, xenografts and syngeneic mouse models. This is due to the large number of works on 2D models over the past five years. In order to broaden anticancer therapy and depict the local tumor microenvironment, it is necessary to overview 3D models. This includes tumor spheroids and organoids. Interest in 3D models is related to their ability to preserve genetic, proteomic, morphological and pharmacotypic tumor features [140].

Tumor spheroids are microsized aggregates of closely packed cells. Organoids are 3D in vitro stem cell-derived cultures [140]. For example, to understand the mechanism of CUDC-907 (compound **2**) cytotoxicity, experiments in study [141] were conducted using a neuroblastoma three-dimensional spheroid. Spheroid neuroblastoma tumors, which mimic the physiological growth characteristics of solid neuroblastoma tumors, were grown using the SH-SY5Y cell line. The results demonstrated significant and dose-dependent inhibition of neuroblastoma tumor growth (reduction in spheroid mass and size under an inverted microscope) in response to CUDC-907 compared to the control. A significant decrease in the number of living cells in the spheroid tumor was observed in response to CUDC-907.

Interestingly, in some cases, the different morphology of the spheroids can help explain the different levels of cytotoxicity of a drug in relation to different types of cells. In another study [142], researchers found that treatment with CUDC-907 (for 6 days) reduced the growth of pleural mesothelioma cells (P31 WT and cisplatin-resistant P31 cis) in 3D spheroids. They also found that the cisplatin-resistant P31 cis cells had a slightly higher sensitivity to CUDC-907 in the 2D cell viability assay (P31 WT IC_50_ was 16 nM and P31 cis was 12 nM). However, this difference became more pronounced when the cells were grown in 3D spheroids, where the cisplatin resistance was more evident. The cell viability was significantly reduced in both cell lines after treatment with 10, 30, and 50 nM of CUDC-907, and the spheroid volume decreased more in the cis-resistant cells. The morphology of the spheroids differed between the two cell lines: P31 WT spheroids were compact and smaller with a smooth surface, whereas the spheroids from the P31 cis line were larger and had an irregular surface. This morphology explains the different cytotoxicity.

Yuan B. et al. [143] established that the dual PI3K/HDAC inhibitor CUDC-907 (compound **2**) significantly inhibited the growth of gallbladder carcinoma (GBC) organoids with minimal toxicity to normal gallbladder organoids. The IC_50_ for CUDC-907 in GBC organoids was found to be between 12.76 and 116.9 nM, according to cell survival curves. Moreover, the expression of phosphorylated AKT was decreased, and the level of acetylated histone H3 was increased after treatment with CUDC-907.

The significantly smaller number of studies using 3D tumor models compared to 2D monolayer cultures is, in our opinion, due to the fact that 2D models require less time and resources and are easier to control. Spheroids and organoids can vary in internal structure, shape, and size for the same cells, making it difficult to obtain statistically significant data. For spheroids, it is also important to select the optimal diameter for each cell type. Additionally, organoids derived from human tissue stem cells are less limited in availability compared to cell lines used for 2D cytotoxicity studies.

## 5. Conclusions

Thus, the development of molecules that target multiple proteins simultaneously is a promising area of research in polypharmacology. These molecules, as demonstrated in the review, show selective cytotoxicity both in vitro and in vivo, have a favorable pharmacokinetic profile, and are less toxic in in vivo studies compared to molecules that target a single protein. Based on the data presented in the review, promising pharmacophore fragments that can be used for the synthesis of these dual-target molecules are aminopyrimidines, aminoquinolines, and sulfonamides for PI3K and ALK; benzimidazoles for ALK; methoxyquinolines and pyrazole carboxamides for AXL; benzodiazepines for HER2; 4-(benzofuran-6-yloxy)-quinazoline moieties for VEGFR; 4-(morpholino)-phenylaminopyrimidines and 4-(piperazin)-phenylaminopyrimidines for FLT3; pyrazolo [3,4]-pyrimidinium moieties for Wee1; aminoquinolinyl moieties for DNMT1; quinolino-5,8-dione for CDC25; aminopyrimidine and 2-(methylamino)thiazol-5-yl moieties for CDK9; 4-(benzo[d]thiazol-5-yl)-5-fluoropyrimidine moiety for DYRK2; and pyrrolopyridone moiety for BRD4. Hydroxamates, hydrazides, benzodiazepines, and *o*-aminobenzamides all act as zinc-binding groups to inhibit HDACs.

It should be noted that a promising area of research in the development of new dual-target molecules is the synthesis and cytotoxicity testing of conjugates of monocarbonyl curcumin analogues, such as 3,5-bis(benzylidene)-4-piperidone, with fragments of the dual-target inhibitors discussed above. The interest in the synthesis of such conjugates stems from their broad effects on carcinogenesis signaling pathways, particularly those involving NF-κB, PI3K/Akt/mTOR, JAK/STAT, and mitochondrial apoptosis [144].

## Figures and Tables

**Table 1:** In vitro data on dual-target PI3K/HDAC inhibitors.

Compound	IC_50_ Against PI3K/HDAC, nM	IC_50_, μM Against Cells	References
**6**	HDAC1 75.5 ± 1.3 HDAC2 70.9 ± 1.2 HDAC3 1.9 ± 0.7 HDAC4,5,6,7,9,11 > 10,000 HDAC8 7498.9 ± 37.2 PI3Kα 2.5 ± 1.1 PI3Kβ 80.5 ± 1.3 PI3Kδ 10.0 ± 0.8 PI3Kγ 57.2 ± 1.3	MV4-11 0.2 ± 0.1 Jeko-1 0.9 ± 0.1 HL60 0.8 ± 0.1 MCF-7 1.5 ± 0.7 HL7702 > 100	[42,44]
ZSTK474 1	PI3Kα 16 PI3Kβ 44 PI3Kδ 5 PI3Kγ 49	MV4-11 2.2 ± 0.3 Jeko-1 1.8 ± 0.2 HL60 1.6 ± 0.2 MCF-7 6.5 ± 2.1 HL7702 > 100	[44]
**7**	PI3Kα 0.59 PI3Kβ 12.69 PI3Kδ 0.59 PI3Kγ 2.02	Jurkat 0.59 K562 4.30 MCF-7 1.98 PC9R 0.38	[45]
GSK2126458	PI3Kα 1.25 PI3Kβ 4.94 PI3Kδ 1.16 PI3Kγ 2.61	Jurkat 0.88 K562 4.36 MCF-7 7.96 PC9R 1.62	

**Table 3:** In vitro data on dual-target AXL/HDAC inhibitors.

Compound	IC_50_ Against AXK/HDAC, nM	IC_50_, μM Against Cells	References
Hit-3 (**12**)	AXL 9.41 ± 0.76 HDAC2 4.06 ± 0.13 HDAC1 7.51 ± 0.64 HDAC3 11.33 ± 0.82 HDAC4 10.95 ± 0.91 HDAC5 13.12 ± 0.76 HDAC6 9.24 ± 0.53 HDAC7 9.54 ± 0.43 HDAC8 8.46 ± 0.22 HDAC9 16.73 ± 0.85 HDAC10 14.56 ± 0.59 HDAC11 12.24 ± 0.56	HCT116 0.07 A549 0.14 MCF-7 0.52 HepG2 0.26 HeLa 0.63 EGFRi-resistant H1975 1.18 AXLi-resistant H460 2.76	[68]
TP0903	AXL 26.23 ± 1.09	Cell viability is less than 70%	
SAHA	HDAC2 9.02 ± 1.81	Cell viability is less than 70%	

**Table 4:** In vitro data on dual-target HER2/HDAC inhibitors.

Compound	IC_50_ Against HER/HDAC, nM	IC_50_, μM Against Cells	References
**13**	HER2 23 ± 1 HDAC1 41 ± 2	HCT-116 9.18 HepG2 6.13 MCF-7 7.86 WI-38 13.95	[74]
Lapatinib	HER2 17 ± 1	–	
Trichostatin A	HDAC1 37 ± 1	–	

**Table 5:** In vitro data on dual-target VEGFR/HDAC inhibitors.

Compound	Degree of Inhibition % at 100 nM	IC_50_, μM Against Cells	References
**14**	VEGFR-2 93.20 ± 1.36 HDAC1 90.24 ± 0.25	HeLa 1.49 ± 0.16 MCF-7 17.86 ± 0.54 A549 4.56 ± 0.33	[84]
SAHA	HDAC1 66.29 ± 1.28	HeLa 1.03 ± 0.33 MCF-7 11.82 ± 0.14 A-549 3.54 ± 0.25	
Fruquintinib	VEGFR-2 89.29 ± 0.62	HeLa 8.35 ± 0.47 MCF-7 > 20 A-549 > 20	
Fruquintinib + SAHA (molar ratio 1:1)	–	HeLa 1.38 ± 0.36 MCF-7 18.50 ± 0.16 A-549 8.12 ± 0.23	

**Table 6:** In vitro data on dual-target FLT3/HDAC inhibitors.

Compound	IC_50_, nM	IC_50_, μM Against Cells	References
**15**	FLT3 39 ± 2.12 HDAC1 17 ± 2.4	MV-4-11 0.061 ± 0.005 Molt4 0.14 ± 0.033 K562 2.75 ± 0.36 Jeko-1 0.25 ± 0.031	[92]
**16**	FLT3 46 ± 1.06 HDAC1 67 ± 5	MV-4-11 0.14 ± 0.017 Molt4 0.90 ± 0.14 Jeko-1 0.33 ± 0.028	
**17**	FLT3 14 ± 0.71 HDAC1 27 ± 1.1	MV-4-11 0.029 ± 0.002 Molt4 0.39 ± 0.052 K562 3.62 ± 0.43 Jeko-1 0.099 ± 0.011 HaCat 80 ± 2.1	
Tandutinib	FLT3 2098 ± 145	MV-4-11 7.63 ± 0.98 Jeko-1 4.96 ± 0.37 HaCat 92 ± 3.4	
SAHA	HDAC1 79 ± 4.5	MV-4-11 3.76 ± 0.26 Molt4 0.32 ± 0.057 K562 7.37 ± 0.91 Jeko-1 0.34 ± 0.043 HaCat 109 ± 9.1	

**Table 7:** In vitro data on dual-target Wee1/HDAC inhibitors.

Compound	IC_50_, nM	IC_50_, μM Against Cells	References
**18**	Wee1 1.2 ± 0.1 HDAC1 196 ± 23 HDAC2 > 2000 HDAC3 156 ± 4 HDAC8 129.5 ± 160 HDAC6 55 ± 1 HDAC10 1310 ± 5 HDAC11 > 2000	MV-4-11 0.076 ± 0.010	[98]
AZD1775	Wee1 1.6 ± 0.2	MV-4-11 0.200 ± 0.016	
SAHA	HDAC1 20 ± 1	MV-4-11 0.643 ± 0.050	

**Table 8:** In vitro data on dual-target DNMT1/HDAC inhibitors.

Compound	IC_50_, nM	IC_50_, μM Against Cells	References
**19**	–	MDA-MB-436 5.03 ± 0.64 MDA-MB-231 4.35 ± 0.71 MDA-MB-468 5.56 ± 0.41 MDA-MB-361 5.51 ± 1.33 MDA-MB-453 1.24 ± 0.17 MDA-MB-157 6.07 ± 0.78 BT-549 8.03 ± 1.17 BT-474 8.82 ± 1.24 HCC1937 9.19 ± 0.45 HCC38 2.11 ± 0.65 HCC1143 4.85 ± 1.59 T-47D 8.29 ± 3.22 MCF-7 > 25.00 4T1 2.06 ± 0.84	[104]
SGI-1027	–	MDA-MB-436 1.21 ± 0.04 MDA-MB-231 0.98 ± 0.12 MDA-MB-468 0.61 ± 0.20 MDA-MB-361 1.32 ± 0.62 MDA-MB-453 0.57 ± 0.12 MDA-MB-157 0.63 ± 0.10 BT-549 1.01 ± 0.34 BT-474 2.09 ± 0.21 HCC1937 0.94 ± 0.07 HCC38 0.59 ± 0.08 HCC1143 1.42 ± 0.18 T-47D 0.96 ± 0.53 MCF-7 1.82 ± 0.42 4T1 1.24 ± 0.77	
SAHA	–	MDA-MB-436 4.01 ± 0.85 MDA-MB-231 2.08 ± 0.21 MDA-MB-468 5.01 ± 1.22 MDA-MB-361 1.59 ± 0.30 MDA-MB-453 1.16 ± 0.20 MDA-MB-157 2.07 ± 0.11 BT-549 7.52 ± 2.37 BT-474 1.79 ± 0.45 HCC1937 10.27 ± 3.56 HCC38 0.86 ± 0.06 HCC1143 1.90 ± 1.18 T-47D 2.82 ± 1.41 MCF-7 19.30 ± 4.71 4T1 1.67 ± 0.75	
**20**	DNMT1 365 DNM3A 103 DNM3B 372 HDAC1 0.20 HDAC6 8.91	MDA-MB-231 0.20 ± 0.01 MDA-MB-468 0.25 ± 0.05 MDA-MB-453 0.17 ± 0.07 MDA-MB-157 0.15 ± 0.01 HCC1937 0.79 ± 0.43 HCC1143 0.12 ± 0.06 HCC38 0.16 ± 0.14 BT-549 0.96 ± 0.39 T-47D 0.05 ± 0.02	[106]
Decitabine	–	MDA-MB-231 > 10 MDA-MB-468 0.031 ± 0.01 MDA-MB-453 > 10 MDA-MB-157 > 10 HCC1937 > 10 HCC1143 > 10 HCC38 1.82 ± 1.32 BT-549 > 10 T-47D > 10	
CM-272	DNMT1 382 DNM3A 85 DNM3B 1200	MDA-MB-231 > 10 MDA-MB-468 0.031 ± 0.01 MDA-MB-453 0.61 ± 0.39 MDA-MB-157 0.12 ± 0.08 HCC1937 0.26 ± 0.03 HCC1143 0.49 ± 0.07 HCC38 0.22 ± 0.14 BT-549 0.21 ± 0.03 T-47D 0.66 ± 0.41	
SAHA	HDAC1 13.87 HDAC6 16.77	MDA-MB-231 3.31 ± 0.82 MDA-MB-468 2.05 ± 1.82	
**21**	DNMT1 202 ± 250 DNMT3A 930 ± 150 DNMT3B 1320 ± 170 HDAC1 4160 ± 190	LS 174 T 3.55 ± 0.75 SW 480 5.49 ± 1.30 Caco-2 3.01 ± 0.25 HCT 116 > 40 HT-29 > 40 DLD-1 > 40 LoVo > 40 SW620 > 40 CT26 > 40 MC38 > 40	[107]
DC-517	–	LS 174 T 2.73 ± 0.05 SW 480 1.20 ± 0.46 Caco-2 2.29 ± 0.26 HCT 116 2.78 ± 0.09 HT-29 3.85 ± 0.75 DLD-1 1.92 ± 0.45 LoVo 2.13 ± 0.53 SW620 2.81 ± 0.05 CT26 2.50 ± 0.47 MC38 2.87 ± 0.07	

**Table 9:** In vitro data on dual-target CDC25/HDAC inhibitors.

Compound	IC_50_, nM	IC_50_, μM Against Cells	References
**22**	–	MDA-MB-231 2.50 ± 0.15	[112]
**23**	CDC25C 230 HDAC1 67.5 HDAC2 415 HDAC3 715.5	MDA-MB-231 1.07 ± 0.07	
**24**	–	MDA-MB-231 2.44 ± 0.29	
MS275	HDAC1 212.5 HDAC2 451.5 HDAC3 898	MDA-MB-231 2.93 ± 0.09	
**25**	–	A172 GBM cells 6.66	[113]
**26**	–	A172 GBM cells 7.04	

**Table 10:** In vitro data on dual-target CDK9/HDAC inhibitors.

Compound	IC_50_, nM	IC_50_, μM Against Cells	References
**27**	CDK9 170 HDAC1 1730 HDAC2 > 50,000 HDAC3 1110	HeLa 1.51 ± 0.24 MDA-MB-231 2.47 ± 0.14 HepG-2 7.28 ± 0.25	[121]
Mocetinostat	ND *	HeLa 18.95 ± 1.84 MDA-MB-231 10.91 ± 0.20 HepG-2 17.13 ± 1.23	
AZD-5438	ND *	HeLa 15.86 ± 1.41 MDA-MB-231 8.04 ± 0.19 HepG-2 3.29 ± 0.52	

* ND—no data.

**Table 11:** In vitro data on dual-target DYRK2/HDAC8 inhibitors.

Compound	IC_50_, nM	IC_50_, μM Against Cells	References
**28**	DYRK2 5.27 ± 0.13 HDAC8 8.06 ± 0.47	SK-HEP-1 0.17 ± 0.01 HuH-7 0.38 ± 0.04 HepG2 0.24 ± 0.02 L02 > 10	[130]
Quisinostat	HDAC8 24.92 ± 2.14	ND *	
YK-2-69	DYRK2 21.55 ± 1.62	ND *	

* ND—no data.

**Table 12:** In vitro data on dual-target BET/HDAC inhibitors.

Compound	IC_50_, nM	IC_50_, μM	References
**29**	BRD4 BD1 420 ± 110 BRD4 BD2 520 ± 180 HDAC1 190 ± 30 HDAC2 360 ± 120 HDAC3 6500 ± 2800	PaTu8988t 2.8 ± 0.2	[139]
**30**	BRD4 BD1 360 ± 130 BRD4 BD2 250 ± 120 HDAC1 110 ± 30 HDAC2 100 ± 20 HDAC3 13,600 ± 4800	PaTu8988t 3.6 ± 0.4	
**31**	BRD4 BD1 370 ± 50 BRD4 BD2 310 ± 50 HDAC1 1300 ± 100 HDAC2 2900 ± 1700 HDAC3 ND *	PaTu8988t 2.6 ± 0.3	
**32**	BRD4 BD1 290 ± 50 BRD4 BD2 180 ± 40 HDAC1 140 ± 50 HDAC2 60 ± 10 HDAC3 11,400 ± 6900	PaTu8988t 4.1 ± 0.7	
CI-944	HDAC1 5000 ± 800 HDAC2 2000 ± 700 HDAC3 11,000 ± 620	PaTu8988t 13.1 ± 2.4	
MS436	BRD4 BD1 2900 ± 400 BRD4 BD2 62,000 ± 15,000 HDAC1 18,800 ± 10,00 HDAC2 > 50,000	PaTu8988t 10.4 ± 1.2	
CI-944+ MS436	-	PaTu8988t 6.0 ± 0.5	

* ND—no data.

## Data Availability

No new data were created or analyzed in this study. Data sharing is not applicable to this article.

## Abbreviations

The following abbreviations and symbols are used in this manuscript:

AKT	Protein kinase B
ALK	Anaplastic lymphoma kinase
AML	Acute myeloid leukemia
AXL	Cell-surface receptor tyrosine kinase
BRD4	Bromodomain-containing protein 4
CDC25	Dual-specificity phosphatase
CDK9	Cyclin-dependent kinase 9
DMSO	Dimethylsulfoxide
DNA	Deoxyribonucleic acid
DNMT	DNA methyltransferase
DYRK2	Dual-specificity tyrosine-phosphorylation-regulated kinase 2
IVIS	In vivo imaging system
FDA	Food and Drug Administration
FLT3	FMS-related tyrosine kinase 3
GBC	Gallbladder carcinoma
HDACs	Histone deacetylases
HER2	Human epidermal growth factor receptor 2
LSD1	Lysine-specific demethylase-1
PI3K	Phosphoinositide-3-kinase
PIP2	Phosphatidylinositol 4,5-bisphosphate
VEGFR	Receptors for vascular endothelial growth factor
